# Shot-Gun Proteomic Analysis on Roots of Arabidopsis *pldα1* Mutants Suggesting the Involvement of PLDα1 in Mitochondrial Protein Import, Vesicular Trafficking and Glucosinolate Biosynthesis

**DOI:** 10.3390/ijms20010082

**Published:** 2018-12-26

**Authors:** Tomáš Takáč, Olga Šamajová, Pavol Vadovič, Tibor Pechan, Jozef Šamaj

**Affiliations:** 1 Centre of the Region Haná for Biotechnological and Agricultural Research, Faculty of Science, Palacký University, Šlechtitelů 27, 78371 Olomouc, Czech Republic; tomas.takac@upol.cz (T.T.); olga.samajova@upol.cz (O.Š.); pavol.vadovic@upol.cz (P.V.); 2 Institute for Genomics, Biocomputing & Biotechnology, Mississippi Agricultural and Forestry Experiment Station, Mississippi State University, Starkville, MS 39759, USA; pechan@ra.msstate.edu

**Keywords:** phospholipase D alpha1, Arabidopsis, proteomics, mitochondrial protein import, quality control, vesicular transport, cytoskeleton

## Abstract

Phospholipase Dα1 (PLDα1) belongs to phospholipases, a large phospholipid hydrolyzing protein family. PLDα1 has a substrate preference for phosphatidylcholine leading to enzymatic production of phosphatidic acid, a lipid second messenger with multiple cellular functions. PLDα1 itself is implicated in biotic and abiotic stress responses. Here, we present a shot-gun differential proteomic analysis on roots of two Arabidopsis *pldα1* mutants compared to the wild type. Interestingly, PLDα1 deficiency leads to altered abundances of proteins involved in diverse processes related to membrane transport including endocytosis and endoplasmic reticulum-Golgi transport. PLDα1 may be involved in the stability of attachment sites of endoplasmic reticulum to the plasma membrane as suggested by increased abundance of synaptotagmin 1, which was validated by immunoblotting and whole-mount immunolabelling analyses. Moreover, we noticed a robust abundance alterations of proteins involved in mitochondrial import and electron transport chain. Notably, the abundances of numerous proteins implicated in glucosinolate biosynthesis were also affected in *pldα1* mutants. Our results suggest a broader biological involvement of PLDα1 than anticipated thus far, especially in the processes such as endomembrane transport, mitochondrial protein import and protein quality control, as well as glucosinolate biosynthesis.

## 1. Introduction

Phospholipases are phospholipid hydrolyzing enzymes with multiple roles in biotic and abiotic stress responses of plants as well as in plant growth and development [1]. Phospholipase D (PLD) alpha 1 (PLDα1) is a member of D subfamily of phospholipases and it shows the highest expression levels among all twelve PLD members in Arabidopsis [2]. Total PLD activity is substantially decreased in Arabidopsis *pldα1* mutants [3]. PLDs utilize preferentially phosphatidylcholine as a substrate, which they hydrolyze in Ca^2+^ dependent manner [2]. This hydrolysis is accompanied with the production of phosphatidic acid (PA), a second messenger bearing important signaling functions [4]. The absence of PLDα1 leads to the reduction of cellular PA pool and membrane lipid remodeling [5,6]. This remodeling affects physical and mechanical properties of membranes leading to endomembrane reorganizations and changes in membrane transport [7,8]. PLDα1 is also involved in the regulation of cytoskeletal dynamics and organization, which is either mediated by PA or by direct binding/association of PLDα1 with the cytoskeleton [3,9,10,11,12]. PLDα1 promotes stomata closure and inhibits their opening [13]. At a molecular level, stomatal movements are governed by PLDα1 through interaction of PA with protein phosphatase 2C (ABI1) [5], NADPH oxidase [14], sphingosine kinase [15] and microtubule associated protein 65-1 [3]. In addition, PLDα1 binds and modulates components of G protein complex during stomatal movements [16,17]. These functions render PLDα1 an important regulator of the plant stress response, growth and development. PLDα1 was shown to be involved in plant response to drought [18], cold [19] and salt stress [12]. This protein has promising biotechnological applications, since its genetic manipulation modulates plant response to abiotic stresses [20]. Nevertheless, PLDs usually act cooperatively (including the production of cellular PA pool), as it was previously exemplified in abscisic acid (ABA)-induced stomatal closure [21]. Arabidopsis mutants of *PLDα1* exhibit conditional phenotypes, whereas under control conditions they show phenotypes similar to the wild-type plants [3,22]. Recent detailed fluorescent in vivo imaging of PLDα1 fused to YFP and expressed in Arabidopsis PLDα1 t-DNA insertion mutants under its own promoter showed that PLDα1-YFP localized to the cytoplasm in the close vicinity of plasma membrane (PM) and exerted developmentally-dependent and tissue-specific expression [12]. Interestingly, most of PLDα1 functions are assigned to processes occurring in leaves. On the other hand, PLDα1 functions in roots are obscure. Shot gun proteomic analysis on genetically modified plants proved to be very useful tool for elucidation of protein functions. Here, we performed a comparative shot gun proteomic analysis on roots of two t-DNA insertion mutants (*pldα1-1* and *pldα1-2*) as compared to the Col-0 wild type. Our results indicated that PLDα1 is involved in mitochondrial protein import and quality control, glucosinolate biosynthesis and that it controls very specific processes of subcellular vesicular transport.

## 2. Results

### 2.1. Overview of Differential Root Proteomes in Two pldα1 Mutants

**Figure 1:**
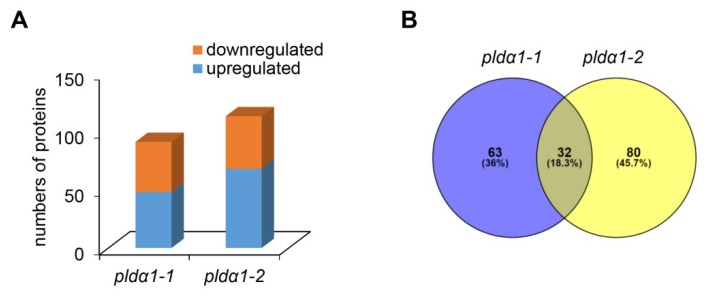
Overview of differential root proteomes of *pldα1* mutants. (**A**) Numbers of proteins with increased and decreased abundances in *pldα1-1* and *pldα1-2* mutant. (**B**) Venn diagram showing difference between differential proteomes the *pldα1-1* and *pldα1-2*.

We carried out a comparative shot-gun proteomic analysis of roots of two *pldα1* mutants compared to the Col-0 as a wild type. First, we compared the number of identified proteins (Figure S1A) and peptides (Figure S1B) in Col-0, *pldα1-1* and *pldα1-2* mutants showing high reproducibility of our analysis (Figure S1A). Considering proteins identified at least in 2 biological replicates, 92%, 82% and 75% of the total proteomes of Col-0, *pldα1-1* and *pldα1-2* roots were found commonly in all three lines (Figure S1C). In *pldα1-1*, 92 proteins with changed abundances were found, while 113 were identified in *pldα1-2* mutant (Figure 1A). In both mutants, 32 proteins were commonly changed (Figure 1B, Table 1). PLDα1 was identified uniquely in the wild type, while we did not detect this protein in two studied mutants, confirming the reliability of our approach. Similarly, we were unable to detect PLDrp1 (PLD regulated protein 1; At5g39570), a phosphoprotein interacting with PLDα1 [23], in *pldα1* mutants. A complete list of all differentially abundant proteins (DAPs) of both mutants is available in Table S1. A detailed outputs of protein identification in all samples is presented in the Supplementary Materials, and deposited in PRoteomics IDEntifications (PRIDE) database (see below).

### 2.2. Classification of Root Differential Proteomes in pldα1 Mutants

**Figure 2:**
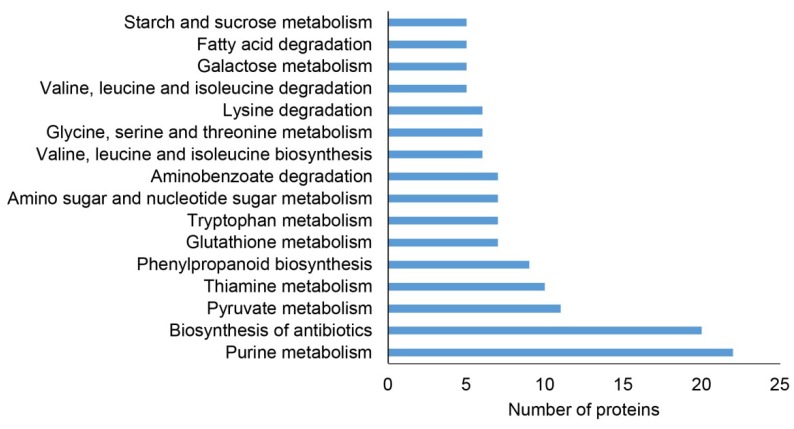
Functional classification of differentially abundant proteins found collectively in roots of *pldα1-1* and *pldα1-2* mutants using Kyoto Encyclopedia of Genes and Genomes (KEGG) pathways analysis.

A Kyoto encyclopedia of genes and genomes (KEGG) pathways analysis is a reasonable tool for the evaluation of proteins involved in metabolism. The highest number of DAPs was classified into the purine metabolism pathway and biosynthesis of antibiotics. Several proteins affected in both mutants are involved in pyruvate metabolism, amino acid biosynthesis and metabolism and phenylpropanoid biosynthesis (Figure 2).

**Figure 3:**
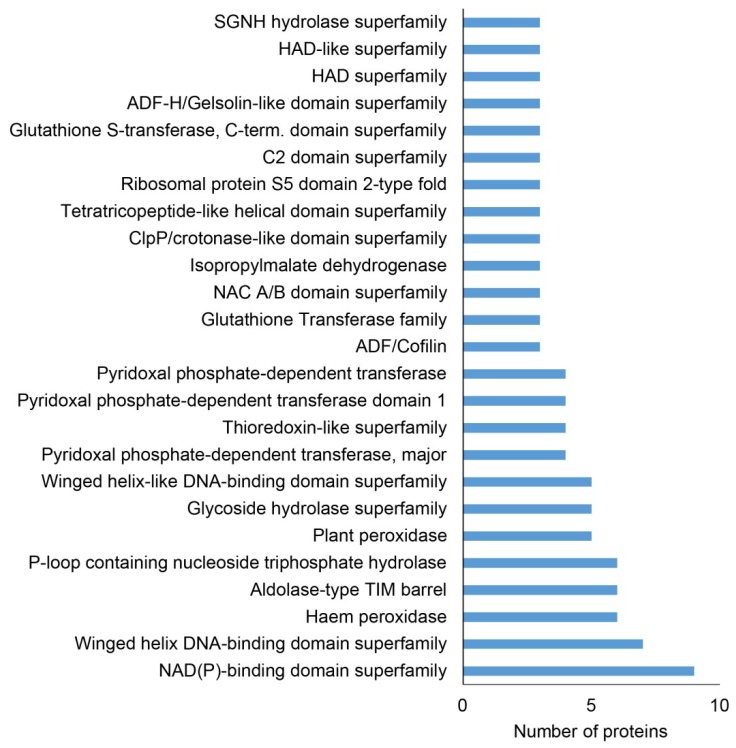
Distribution of protein families, in differentially abundant proteins found collectively in roots of *pldα1-1* and *pldα1-2* mutants, as evaluated by InterPro application of Blast2Go software. HAD = haloacid dehydrogenase; ADF = actin depolymerization factor; TIM = mitochondrial import inner membrane translocase; NAC = nascent polypeptide-associated complex; SGNH = serin, glycin, asparagine, histidin.

Additionally, we screened differential proteomes of both mutants for the abundance of protein families, as evaluated by the Blast2Go software using InterPro application (Figure 3, Table S2). We identified nine proteins belonging to the NAD(P) binding protein superfamily, while seven proteins belonged to the Winged helix DNA-binding domain superfamily. Later ones include proteins with different functions (Table S2) and possessed specific DNA binding mechanisms different from sequence specific binding. They display an exposed patch of hydrophobic residues implicated in protein-protein interactions [24]. The peroxidases and aldolase-type TIM (triose phosphate isomerase) barrel protein family represented abundant protein classes found in both *pldα1* mutants (Figure 3, Table S2). These proteins might show higher sensitivity to PLDα1 and PA deregulation in Arabidopsis.

Furthermore, we classified differential proteomes of *pldα1* mutants (combined) using a gene ontology (GO) annotation analysis. The highest number of the DAPs was assigned to metabolic processes and nitrogen compound metabolic processes. A significant number of DAPs were involved in response to stress as well as establishment of localization (Figure S2A). Higher levels of GO revealed that proteins annotated as involved in stress response belong to GO class called response to osmotic stress (Table S3). Concerning cellular compartment, the GO ontology analysis showed that the highest number of DAPs was assigned to cytosol, followed by plastid, mitochondria, protein complex and the nucleus (Figure S2B).

**Figure 4:**
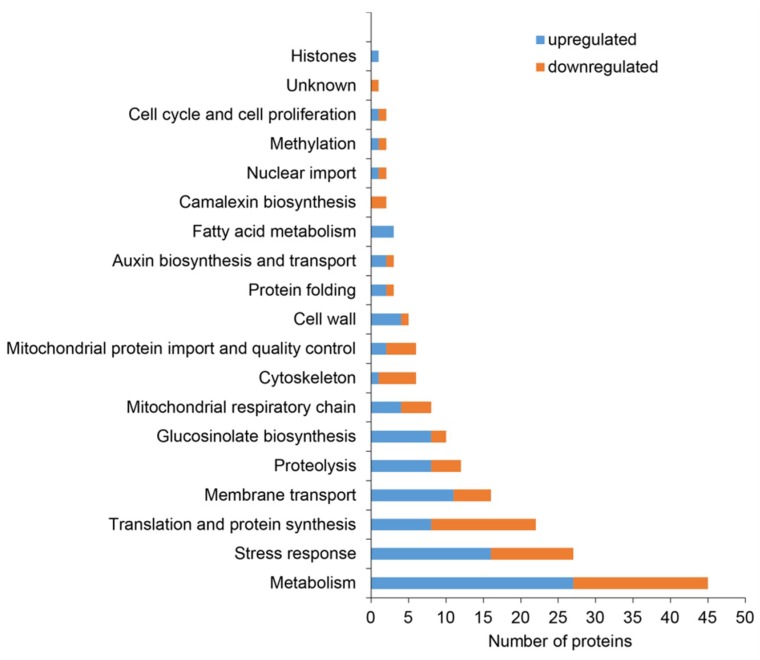
Functional classification of differentially abundant proteins found collectively in roots of *pldα1-1* and *pldα1-2* mutants based on published information, as presented in Table S1.

Since GO ontology analysis does not consider all relevant information about protein functions, we decided to classify the combined differential proteome based on published data (Figure 4, Table 1 and Table S1).

**Figure 5:**
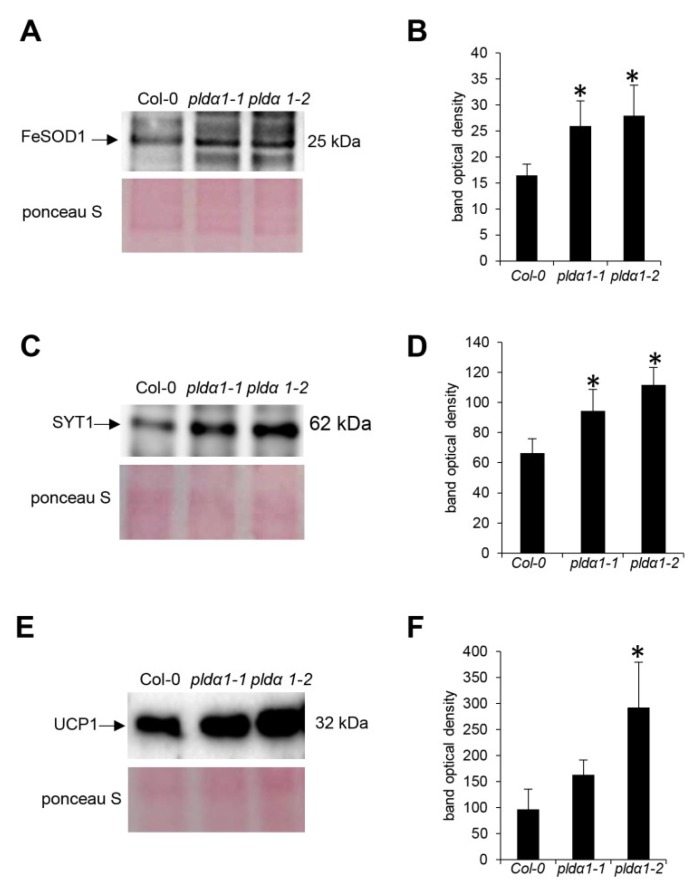
Immunoblotting analysis of ironic superoxide dismutase 1 (FeSOD1), syntaptotagmin 1 (SYT1) and mitochondrial uncoupling protein 1 (UCP1) in the roots of the Arabidopsis wild type and *pldα1* mutants. (**A**,**C**,**E**) Immunoblots probed with anti-FeSOD (**A**), anti-SYT1 (**B**) and anti-UCP1 (**C**) antibodies and visualization of proteins transferred on nitrocellulose membranes using Ponceau S. (**B**,**D**,**F**) Optical density quantification of the respective bands in (**A**,**C**,**E**). Stars indicate significant differences between mutants and wild type at *p* ≤ 0.05 according to the Student t-test. Error bars represent standard deviations.

Apart from the high number of DAPs with diverse metabolic functions, proteins related to the stress response were the second most abundant category (Figure 4, Table S1). Notably, PLDα1 deficiency in both mutants negatively affected the abundance of protein C2-domain ABA-related 10 (CAR10), a component of the pyrabactin resistance1/pyrabactin resistance1-like/car (PYR/PYL/CAR) receptors for ABA [25]. Additionally, we noticed the significant disturbance of antioxidant defense and redox homeostasis. This is represented by the increased abundance of ironic superoxide dismutase 1 (FeSOD1), ascorbate peroxidase and peptide methionine sulfoxide reductase B6. Secretory peroxidases exhibited varying changes in protein abundance, while catalase and glutathione S-transferase F7 had a lower abundance in the mutants compared to the wild type. To prove the increased abundance of FeSOD1, we performed an immunoblotting analysis on *pldα1* mutants using anti-FeSOD1 polyclonal primary antibody (Figure 5A,B). The *Arabidopsis thaliana* genome contains three isoforms of FeSOD, out of which FeSOD2 and FeSOD3 are not expressed in the roots. Therefore, anti-FeSOD antibody recognizes FeSOD1 in the Arabidopsis roots. These analyses showed significant upregulation of FeSOD1 abundance in both *pldα1* mutants.

Interestingly, PLDα1 deficiency also leads to deregulation of proteins involved in cell wall remodeling (Figure 4, Table S1), which represents one of the primary plant defense responses to pathogens. This is consistent with the known role of PLDα1 in plant biotic stress [26]. Furthermore, we have found several defense related proteins differentially abundant in the *pldα1* mutants, including secretory peroxidases, nitrile specifier protein 1 and defensin-like protein 1 (Table S1). The majority of these proteins show increased abundance in the mutants. Notably, proteins involved in glucosinolate biosynthesis (discussed below) are highly represented, showing mostly increased abundances in the mutants (Figure 4).

**Figure 6:**
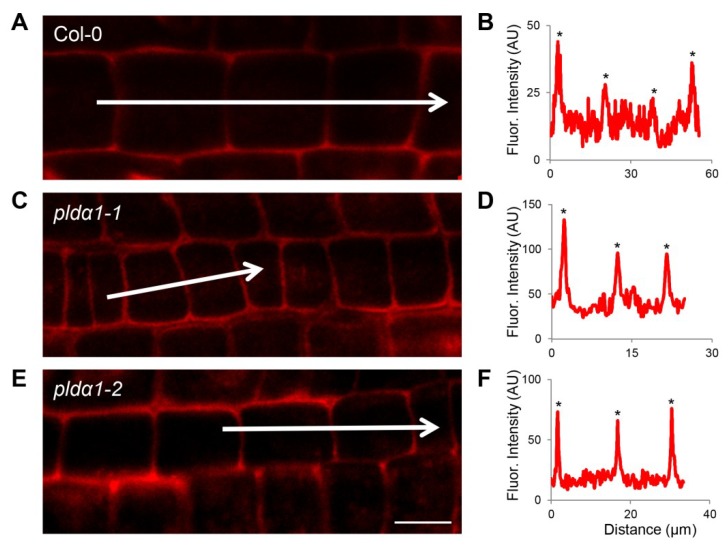
Immunolocalization of synaptotagmin (SYT1) in root epidermal cells of wild type (**A**), *pldα1-1* (**C**) and *pldα1-2* (**E**). (**B**,**D**,**F**) Fluorescence intensity profiles of immunolabeled synaptotagmin distributions in wild type (**B**), *pldα1-1* (**D**) and *pldα1-2* (**F**). Arrows indicate positions of measured cells for fluorescence intensity profiles. Asterisks indicate peaks of highest fluorescence intensities in measured cells. Note that fluorescence intensities in *pldα1* mutants are much higher in comparison to the wild type, indicating overabundance of SYT1 in these mutants. Scale bar = 10 μm.

Additionally, we have found numerous proteins involved in membrane fusion and transport. They are described in detail in the Discussion section. Among others, a PLDα1 deficiency resulted in accumulation of synaptotagmin 1 in the mutants. These proteomic data were successfully validated using immunoblotting analyses (Figure 5C,D) and immunolocalization of the syntaptotagmin 1 (SYT1) protein in intact roots showing an increased accumulation in both *pldα1* mutants (Figure 6).

**Figure 7:**
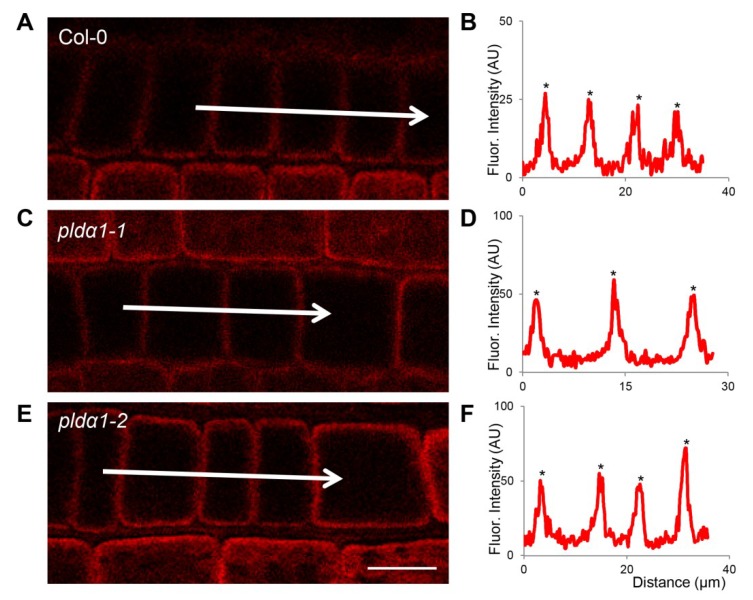
Immunolocalization of mitochondrial uncoupling protein 1 (UCP1) in root epidermal cells of wild type (**A**), *pldα1-1* (**C**) and *pldα1-2* (**E**). (**B**,**D**,**F**) Fluorescence intensity profiles of immunolabeled synaptotagmin distributions in wild type (**B**), *pldα1-1* (**D**) and *pldα1-2* (**F**). Arrows indicate positions of measured cells for fluorescence intensity profiles. Asterisks indicate peaks of highest fluorescence intensities in measured cells. Note that fluorescence intensities in *pldα1* mutants are much higher in comparison to the wild type, indicating an overabundance of UCP1 in these mutants. Scale bar = 10 μm.

Proteins involved in ribosome biogenesis and translation, mitochondrial respiration, mitochondrial protein import and quality control represented a significant functional classes altered by PLDa1 deficiency (Figure 4, Table 1 and Table S1). These findings might indicate defects of cytosolic translation and mitochondrial protein import resulting in changed abundances of mitochondrial proteins. Therefore we searched for proteins carrying mitochondrial targeting signal among DAPs. We have found 19 proteins with varying changes in their abundance, suggesting an altered homeostasis in the import of mitochondrial proteins (Table S4). One of such proteins, mitochondrial uncoupling protein 1 (UPC1) has increased abundance in the mutants, being in agreement with the immunoblotting analysis (Figure 5E,F) and immunolocalization of uncoupling protein 1 (UCP1) protein (Figure 7). In addition, we observed also decreased levels of MORF8 (multiple site organellar RNA editing factor, designated also as RIP1; Table S1), a protein important for mitochondrial mRNA editing. Finally, absence of PLDα1 in both mutants affects also a cluster of components of the mitochondrial respiratory chain. Thus, PLDα1 is likely required for multiple mitochondrial functions in Arabidopsis (Table S1, Figure 4).

PLDα1 and PA are important regulators of actin and microtubule cytoskeletons in plants [11,27]. As expected, PLDα1 deficiency in both mutants resulted in differential abundances of actin and microtubule associated proteins, including actin1 and actin depolymerizing factors (ADFs) 1, 8 and 10 (showing decreased abundances in *pldα1* mutants) (Table 1 and Table S1). Such results indicate possible disturbances in actin monomer turnover and actin polymerization in *pldα1* mutants. Additionally, we identified two protein candidates potentially important for microtubule regulation by PLDα1. Both proteins were detected uniquely in *pldα1* mutants and are involved in tubulin monomer folding. Tubulin-folding cofactor B is a member of the Arabidopsis pilZ domain proteins [28,29]. It interacts with alpha-tubulin and its overexpression results in reduced number of microtubules [30]. Chaperone prefoldin 6 is required for tubulin monomer abundance, microtubule dynamics and organization [31].

## 3. Discussion

This differential proteomic analysis on roots of *pldα1* mutants revealed that PLDα1 is required for homeostasis of proteins involved in diverse processes. In this study, we focused especially on potential new functions of PLDα1 such as mitochondrial protein import and quality control, vesicular trafficking and glucosinolate biosynthesis. Considering the regulatory and catalytic roles of PLDα1, we assume that besides its lipid hydrolyzing activity, the changes in the proteomes of *pldα1* mutants occurred as a consequence of compromised PA, G protein complex and ABA signalling.

### 3.1. New Insights into ABA Signalling

PLDα1 derived PA is a crucial regulator of stomatal movements, because it targets/binds multiple proteins essential for this process, including ABI1 [5], NADPH oxidase [14], G protein complexes [13] and MAP65-1 [3]. Assuming from our results, there seems to be a broader impact on other components of ABA signalling because PLDα1 deficiency negatively affected the abundance of protein C2-DOMAIN ABA-RELATED 10 (CAR10). CAR10 interacts with PYR/PYL ABA receptors and recruits them transiently into phospholipid vesicles, thus positively regulating ABA signaling [25]. The PYR/PYL/CAR receptors also bind to ABI1 [32]. These data indicate a possible feedback regulation of CAR10 abundance in the absence of PLDα1 and decreased levels of PA. In addition, aquaporins PM intrinsic protein 1-2 (PIP1-2) and PIP2-1 are ABA-inducible proteins, which promote water uptake and transport [33], and they bind PA [34], PLDδ and PLDγ [35]. Our proteomic analysis showed that abundances of these proteins substantially increased in *pldα1* mutants.

### 3.2. Mitochondrial Protein Import and Quality Control

According to our results, PLDα1 deficiency in mutants caused a deregulation of proteins involved in protein import to mitochondria, including mitochondrial import inner membrane translocase subunits TIM23-2 and TIM13, which are downregulated. While TIM23-2 is a translocase responsible for the transport of mitochondrial precursor proteins carrying a cleavable N-terminal pre-sequence [36], TIM13 is a member of small TIM complex delivering client precursors that pass through the TOM (mitochondrial import outer membrane translocase) channel to Tim22 in the mitochondrial intermembrane space [37]. Therefore, the import of nucleus-encoded mitochondrial proteins is altered in *pldα1* mutants. Along with altered protein import to mitochondria, PLDα1 deficiency may affect also N-terminal presequence cleavage (inferred by increased abundance of presequence protease 1 in *pldα1* mutants) occurring after protein precursor import into mitochondria [38]. Furthermore, we provided experimental evidence on deregulation of prohibitin 6 involved in mitochondrial protein folding [39]. Prohibitins (PHBs) are considered to be structural proteins that form a scaffold-like structure for interacting with a set of proteins involved in various mitochondrial processes [39]. These proteins participate in the assembly of multi-subunit complexes such as mitochondrial respiratory complex [40]. Accordingly, several proteins of the mitochondrial electron transport chain show significant changes in their abundance in both mutants as compared to the wild type. Mitochondrial protein import machinery was also reported to be in close interaction with the organization of respiratory complexes. Tim23-2 is localized also in respiratory complex 1 and its genetic modification leads to altered transcription of mitochondrial proteins and defective mitochondria biogenesis [36]. A similar role in mitochondria biogenesis was found for prohibitins [41]. Thus far, PLDα1 was not linked to these mitochondrial functions, although the ATP synthase subunit gamma and ADP/ATP carrier protein were targeted by PA in Arabidopsis [34].

### 3.3. Vesicular Transport

PLD-derived PA can regulate membrane transport by direct modification of membrane curvature or by recruiting important regulatory proteins [42]. These proteins positively affect protein internalization [43,44], vesicle fusion and aggregation [45]. In Drosophila, PLD activity couples endocytosis with retromer dependent recycling [46]. Our findings indicate that PLDα1 alters multiple sites of endomembrane system. For example, in both mutants we detected decreased abundances of vacuolar H^+^ ATPases (subunits D and d2), which control multiple events in endomembrane transport by acidification of endomembrane compartments [47].

In accordance with the known involvement of PLDs in vesicle fusions, we observed an increased abundance of alpha-soluble N-ethylmaleimide-sensitive factor (NSF) attachment protein 2 (Alpha-SNAP2) in the *pldα1-1* mutant. Alpha-SNAP proteins bind the soluble NSF attachment protein receptor (SNARE) complex [48] and are required for the vesicle pre-docking, an initial step of the membrane fusion reaction [49,50]. The precise function of alpha-SNAP2 is unknown, though it might require PLDα1. Remarkably, alpha-SNAP2 interacting syntaxin 32 (SYP32), a Golgi localized Qa SNARE [51] was found as upregulated in *pldα1* mutants. Thus, PLDα1 might be necessary for SNARE-SNAP protein complexes stability.

We identified several proteins involved in the endocytic pathway as differentially regulated in both *pldα1* mutants. These include mainly the probable clathrin assembly protein At4g32285 (not detected in *pldα1* mutants), which is involved in clathrin-mediated endocytosis [52]. Clathrin assembly proteins interact directly with proteins of the clathrin coat and are able to bind phospholipids [53]. Two such proteins were identified as PA-binding proteins [34]. Furthermore, PLDα1 localized in the vicinity of clathrin heavy chain and microtubules of Arabidopsis root cells [12] and it may directly bind clathrin in a complex containing adaptor protein-2 (AP-2) [54]. Vacuolar protein sorting 29 (VPS29), a protein found uniquely in the *pldα1-1* mutant, is a component of retromer complex. This is a coat complex localized to the cytosolic face of endosomes and involved in intracellular sorting of some transmembrane proteins [55]. VPS29 is important for normal morphology of prevacuolar compartment (PVC) and plays crucial role in recycling vacuolar sorting receptors from the PVC to the *trans* Golgi network (TGN) during trafficking of soluble proteins to the lytic vacuole [56,57]. These data uncovers new endocytic proteins affected in *pldα1* mutants.

PLDα1 deficiency in both mutants altered also abundances of proteins involved in the regulation of endoplasmic reticulum (ER) to Golgi transport. Protein transport protein SEC13 homolog A is upregulated nearly threefold in both *pldα1* mutants. Sec13 makes a lattice structure together with Sec31 to form COPII vesicles [58], which are responsible for ER to Golgi transport. According to our results, PLDα1 may have also an impact on the morphology of Golgi apparatus, inferred by the upregulation of Golgin candidate 5 (also known as the TATA element modulatory factor) in the *pldα1-2* mutant [59,60]. Another protein important for ER to Golgi trafficking is vesicle-associated protein 1-2 (PVA12, also known as VAP27-3), which is upregulated in the *pldα1-1* mutant. This is an ER-localized protein belonging to a VAP27 family [61]. It binds to oxysterol-binding protein-related protein 3B [62], which is also upregulated in the mutants and is proposed to cycle between the ER and the Golgi [62]. Recently, PVA12 was shown to colocalize and interact with Networked 3C (NET3C) at ER–PM contact sites [61]. Considering PLDα1 localization in the PM vicinity, we suggest an involvement of this protein in ER-PM attachment. This is emphasized by an increased abundance of synaptotagmin 1 (SYT1) in *pldα1* mutants, representing a protein mediating the ER-PM contacts in Arabidopsis [63].

PLDα1 depletion leads to changed abundance of proteins regulating the membrane transport. Changes in protein level might be a result of deregulation of protein synthesis and proteolysis or transcriptional control. Previously, it was shown that changes in membrane transport might result in changed abundance of proteins. This was exemplified for example in Arabidopsis roots exposed to brefeldin A (BFA), which blocks secretion/exocytosis by aggregation of TGN and PM-derived vesicles surrounded by Golgi stacks into so called BFA-compartments [64]. Altered endocytosis and vacuolar trafficking by wortmannin lead to altered abundances of vacuolar proteases potentially leading to defected protein degradation [65]. Similar downregulation of such protease, subtilisin-like protease SBT1.7 is encountered also in roots of *pldα1* mutants. Based on our proteomic data we suggest that this dynamics of membrane transport regulatory processes might result from defected protein degradation and as a feedback mechanism of PLDα1 depletion-induced changes in membrane architecture, membrane transport and PA accumulation.

### 3.4. Glucosinolate Biosynthesis

PLDs have been shown to crosstalk with hormonal signaling in plants. In addition to their well-known role in ABA signaling, they also participate in salicylic acid signaling by controlling relocation of NPR1, an essential regulator of SA induced gene transcription, into the nucleus [66]. In addition, PLDs might be activated by cytokinins [67] and ethylene [68]. Constitutive triple response 1 (CTR1), a negative regulator of ethylene response is a potential target of PA [69]. PLDs are also involved in auxin distribution. Thus, PLDζ-derived PA is required for protein phosphatase 2Ac (PP2Ac) recruitment to the membrane resulting in altered auxin efflux carrier component 1 (PIN1) phosphorylation and polar distribution [7]. Auxins share an initial steps of biosynthetic pathway with glucosinolates [70,71]. Arabidopsis mutants with reduced glucosinolate contents show severe auxin phenotypes [72]. Generally, glucosinolates are secondary messengers produced in *Brassicaceae* with important defense and developmental functions [70,73]. PLDα1 deficiency in mutants causes increased abundances of enzymes involved in glucosinolate biosynthesis, including four subunits of 3-isopropylmalate dehydrogenase and methylthioalkylmalate synthase, all involved in the chain elongation machinery. Enzymes involved in the biosynthesis of the core glucosinolate structure, namely cytochrome P450 83B1, glutathione S-transferase F9, indole glucosinolate O-methyltransferase 1 and adenylyl-sulfate kinase 1, showed similar trends in their abundances (Table S1). PLDα1 induced an imbalance of indole glucosinolate o-methyltransferase 1 abundance, which is a glucosinolate modifying enzyme [71]. Glutathione synthase 1 showed an increased abundance in mutants, most likely contributing to the glutathione pool, which serves as a sulfur donor within the second stage of GLS biosynthesis [71]. Such differential regulation of enzymes involved in one metabolic pathway in untargeted proteomic approach is very unusual, suggesting that PLDα1 might be a master regulator of glucosinolate biosynthesis. It is likely that this regulation is mediated via PA, since cytochrome P450 83B1 is a PA-binding protein, as identified in a proteomic screen [34].

## 4. Materials and Methods

### 4.1. Plant Material

Seeds of *Arabidopsis thaliana* wild type (ecotype Col-0) as well as *pldα1-1* (SALK_067533) and *pldα1-2* (SALK_053785) t-DNA insertion mutants were used in this study. Following ethanol surface-sterilization, they were cultivated vertically on solid half-strength Murashige-Skoog (MS) media at 21 °C under 16/8 light/dark illumination conditions for 14 days. Roots were quickly dissected and harvested for protein extraction. Proteomic analyses were performed in four biological replicates. Roots of 30 seedlings were pooled in one biological replicate.

### 4.2. Protein Extraction and Trypsin Digestion

Samples were ground in liquid nitrogen and subjected to phenol protein extraction followed by ammonium acetate/methanol precipitation as described by Takáč et al. [74]. Cleaned precipitates were dissolved in 6 M urea in 100 mM Tris (pH 7,8). Prior to trypsin digestion, extracts containing 50 µg of proteins (in volume of 50 µl) were diluted with 100 mM Tris-HCl (pH7,8) to adjust the urea concentration bellow 1 mM. Proteins were digested with trypsin (Promega;1 µg of trypsin to 50 µg of proteins) at 37 °C overnight. Reaction was stopped by addition of 4 µL of acetic acid. Peptide mixtures were cleaned using C18 gravity flow cartridges (Bond Elut C18; Agilent Technologies, Santa Clara, CA, USA) according to manufacturer’s instructions. Peptides eluted by 95% acetonitrile were dried using vacuum concentrator and stored under −80 °C until analysis.

### 4.3. Liquid Chromatography, Mass Spectrometry, Protein Identification and Relative Quantitative Analysis

Liquid chromatography-MSMS and protein identification was performed as published previously [74] with minor modifications. As target database and decoy databases, the UNIPROT (www.uniprot.org) Arabidopsis genus taxonomy reviewed protein database (17,586 entries as of 31st September 2017), and its reversed copy (created automatically by the software) were used, respectively. The mass spectrometry proteomics data have been deposited to the ProteomeXchange Consortium via the PRIDE partner repository with the dataset identifier PXD011196.

The quantitative analysis was done using the ProteoIQ 2.1 (NuSep Inc., Athens, GA, USA) software as published previously [75]. The ANOVA *p* ≤ 0.05 was used to filter statistically significant results. Proteins with fold changes higher than 1.5 were considered as differentially abundant. Proteins present at least in two biological replicates and identified by at least two peptide spectral matches were quantified.

### 4.4. Bioinformatic Analysis

Gene ontology (GO) annotation analysis of DAPs was performed using Blast2Go software [76]. BLAST searching was performed against the *Arabidopsis thaliana* NCBI database allowing 1 BLAST Hit. The annotation was carried out by using these parameters: E Value Hit filter: 1.0 × 10^−6^; Annotation cut off: 55; GO weight: 5. The prediction of presence of mitochondrial targeting pre-sequence in differential proteomes of both mutants was performed using MitoFates [77].

### 4.5. Immunoblotting Analysis

Immunoblotting analysis was performed on protein extracts derived from roots of 14 day-old plants of wild type, as well as *pldα1-1* and *pldα1-2* mutants following published procedure [74]. Anti-synaptotagmin (PhytoAb; dilution 1:1000), anti-FeSOD (Agrisera; dilution 1:3000) and anti-UCP1 (Agrisera; dilution 1:1000) primary antibodies were used. Immunoblot analyses were carried out in three biological replicates. Differences in signal intensity between wild type and the mutants were statistically evaluated using Student’s *t*-test (*p* < 0.05).

### 4.6. Whole Mount Immunofluorescence Labelling

Immunolocalization of SYT1 and UCP1 proteins in root wholemounts was carried out as published previously [78]. As primary antibodies, we have used the rabbit anti-synaptotagmin 1 antibody (PhytoAb; 1:200) and anti-UCP1 antibody (Agrisera; 1:200), while Alexa-Fluor 647 goat anti-rabbit IgG was exploited as secondary antibody. Microscopic observations were performed using the Zeiss 710 Confocal Laser Scanning Microscope platform (Carl Zeiss, Jena, Germany), using excitation lines at 405 and 561 nm from argon, HeNe, diode and diode pumped solid-state lasers. ZEN 2010 software (Carl Zeiss) was used for post-processing, default deconvolution and quantification of fluorescence intensity. Additionally, Photoshop 6.0/CS, and Microsoft PowerPoint softwares were used to process the obtained images.

## 5. Conclusions

Based on this proteomic analysis, PLDα1 is a protein which in addition to its well-known functions in ABA signalling and cytoskeleton organization, important for the homeostasis of proteins involved in mitochondrial protein import, vesicular trafficking and glucosinolate biosynthesis.

## Figures and Tables

**Table 1:** List of differentially abundant proteins found commonly in roots of both *pldα1-1* and *pldα1-2* mutants as compared to the wild type (WT). n.a. = not applicable.

TAIR Accession Number	UNIPROT Accession Number	Sequence Name	*pldα1-1*/Col-0 Ratio	*pldα1-2*/Col-0 Ratio	*pldα1-1*/Col-0 *p* Value	*pldα1-2*/Col-0 *p* Value
		**Translation**				
Q8LD46	At2g39460	60S ribosomal protein L23a-1	20.82	7.62	0.01	0.012
Q9LHG9	At3g12390	Nascent polypeptide-associated complex subunit alpha-like protein 1	1.82	1.91	0.052	0.029
Q9FJH6	At5g60790	ABC transporter F family member 1	Unique in WT	0.38	n.a.	0.03
		**Stress response**				
P50700	At4g11650	Osmotin-like protein OSM34	0.42	0.26	0.048	0.039
Q9LYW9	At5g03160	DnaJ protein P58IPK homolog	4.03	4.11	0.004	0.026
P24102	At2g38380	Peroxidase 22	1.79	1.99	0.031	0.005
Q9LSY7	At3g21770	Peroxidase 30	Unique in mutant	Unique in mutant	n.a.	n.a.
P42760	At1g02930	Glutathione S-transferase F6	0.29	0.26	0.049	0.029
Q9SRY5	At1g02920	Glutathione S-transferase F7	0.31	0.25	0.053	0.036
Q38882	At3g15730	Phospholipase D alpha 1	Unique in WT	Unique in WT	n.a.	n.a.
Q9FKA5	At5g39570	Uncharacterized protein At5g39570 (PLD regulated protein1, PLDRP1)	Unique in WT	Unique in WT	n.a.	n.a.
P32961	At3g44310	Nitrilase 1	1.79	1.79	0.018	0.023
		**Membrane transport**				
Q9SRI1	At3g01340	Protein transport protein SEC13 homolog A	2.55	2.97	0.001	0.011
Q8S9J8	At4g32285	Probable clathrin assembly protein At4g32285	Unique in WT	Unique in WT	n.a.	n.a.
		**Mitochondrial respiratory chain**				
Q9FT52	At3g52300	ATP synthase subunit d, mitochondrial	1.69	1.57	0.047	0.046
O81845	At3g54110	Mitochondrial uncoupling protein 1	1.67	2.14	0.02	0.01
P93306	AtMg00510	NADH dehydrogenase [ubiquinone] iron-sulfur protein 2	0.50	0.56	0.028	0.054
Q9S7L9	At1g22450	Cytochrome c oxidase subunit 6b-1	Unique in mutant	Unique in mutant	n.a.	n.a.
		**Glucosinolate biosynthesis**				
O49340	At2g30750	Cytochrome P450 71A12	Unique in WT	Unique in WT	n.a.	n.a.
Q9FG67	At5g23010	Methylthioalkylmalate synthase 1, chloroplastic	1.13	1.74	0.01	0.036
		**Other functions**				
Q9LSB4	At3g15950	TSA1-like protein	1.33	1.71	0.049	0.003
Q9SP02	At5g58710	Peptidyl-prolyl cis-trans isomerase CYP20-1	1.13	1.56	0.004	0.013
Q8VYV7	At5g66120	3-dehydroquinate synthase, chloroplastic	0.39	0.44	0.046	0.01
Q9AV97	At1g79500	2-dehydro-3-deoxyphosphooctonate aldolase 1	Unique in mutant	Unique in mutant	n.a.	n.a.
Q9FHR8	At5g43280	Delta(3,5)-Delta(2,4)-dienoyl-CoA isomerase, peroxisomal	0.44	0.30	0.011	0.003
Q9FIK7	At5g47720	Probable acetyl-CoA acetyltransferase, cytosolic 2	0.59	1.71	0.042	0.055
Q9FLQ4	At5g55070	Dihydrolipoyllysine-residue succinyltransferase component of 2-oxoglutarate dehydrogenase complex 1, mitochondrial	8.28	5.54	0.008	0.029
Q9FMT1	At5g14200	3-isopropylmalate dehydrogenase 3, chloroplastic	1.49	1.61	0.002	0.01
Q9LQ04	At1g63000	Bifunctional dTDP-4-dehydrorhamnose 3,5-epimerase/dTDP-4-dehydrorhamnose reductase	1.38	1.60	0.038	0.029
Q9SA14	At1g31180	3-isopropylmalate dehydrogenase 1, chloroplastic	1.52	1.55	0.019	0.02
Q9SIU0	At2g13560	NAD-dependent malic enzyme 1, mitochondrial	4.99	2.10	0.011	0.009

## Supplementary Materials

Supplementary materials can be found at http://www.mdpi.com/1422-0067/20/1/82/s1.
