# Supplementary material for: Shot-Gun Proteomic Analysis on Roots of Arabidopsis pldα1 Mutants Suggesting the Involvement of PLDα1 in Mitochondrial Protein Import, Vesicular Trafficking and Glucosinolate Biosynthesis
Source: Int J Mol Sci. 2018 Dec 26;20(1):82. doi: 10.3390/ijms20010082 (PMC6337374; doi:10.3390/ijms20010082)
Supplement: Supplementary file 1 [file ijms-20-00082-s001.zip › Supplementary Tables and Figures.pdf]

Supplementary Material

Shot-Gun Proteomic Analysis on Roots of Arabidopsis Pldα1 Mutants Suggesting involvement of Pldα1 in Mitochondrial Protein Import, Vesicular Trafficking and Glucosinolate Biosynthesis

Tomáš Takáč[1], Olga Šamajová[1], Pavol Vadovič[1], Tibor Pechan[2], Jozef Šamaj[1]*

*** Correspondence:** Jozef Šamaj, jozef.samaj@upol.cz

Table S1. Quantification details of proteins identified in roots of Arabidopsis *pldα1-1* and *pldα1-2* mutant seedlings. n.a. = not applicable

Protein accession			fold change		p value					
TAIR	UNIPROT	Sequence Name	*pldα1-1* vs Col-0	*pldα1-2* vs Col-0	*pldα1-1* vs Col-0	*pldα1-2* vs Col-0	Total Score	Total Peptides	Total % Seq Coverage	Total Spectra
		**Stress response**								
AT2G01540	Q9ZVF1	CAR10_ARATH Protein C2-DOMAIN ABA-RELATED 10	Unique in Col-0		n.a.		9.95	3	26.67	3
AT2G45960	Q06611	PIP12_ARATH Aquaporin PIP1-2	2.77		0.046		14.41	3	27.27	13
AT3G53420	P43286	PIP21_ARATH Aquaporin PIP2-1	4.66		0.036		8.42	2	18.12	9
AT1G20450	P42759	ERD10_ARATH Dehydrin ERD10		0.67		0.032	8.60	2	15.00	4
At4g16260	Q8VZJ2	BGNEM_ARATH Probable glucan endo-1,3-beta-glucosidase At4g16260		0.59		0.003	51.17	11	55.81	125
AT2G02130	Q9ZUL7	DEF01_ARATH Defensin-like protein 1	2.01		0.045		10.88	2	31.17	17
AT4G11650	P50700	OSL3_ARATH Osmotin-like protein OSM34	0.42	0.26	0.048	0.036	9.91	2	16.39	20
At5g03160	Q9LYW9	DNJ36_ARATH DnaJ protein P58IPK homolog	4.03	4.11	0.004	0.026	7.56	2	7.88	5
At2g24580	Q9SJA7	SOX_ARATH Probable sarcosine oxidase		2.35		0.051	15.55	4	19.47	12
AT5G39570	Q9FKA5	Y5957_ARATH Uncharacterized protein At5g39570 (PLD REGULATED PROTEIN1, PLDRP1)	Unique in Col-0	Unique in Col-0	n.a.	n.a.	8.98	2	9.19	8
At3g10850	O24496	GLO2C_ARATH Hydroxyacylglutathione hydrolase cytoplasmic		1.99		0.013	8.55	2	16.28	12
At5g45510	Q8VZC7	DRL36_ARATH Probable disease resistance protein At5g45510		Unique in *pldα1-2*		n.a.	7.23	2	3.85	4

AT3G16400	Q9SDM9	JAL28_ARATH Nitrile-specifier protein 1		1.49		0.013	90.92	21	54.89	161
AT4G25100	P21276	SODF1_ARATH Superoxide dismutase [Fe] 1, chloroplastic		2.58		0.03	10.06	2	22.17	18
AT4G08390	Q42592	APXS_ARATH L-ascorbate peroxidase S, chloroplastic/mitochondrial	2.25		0.045		25.21	6	33.33	25
AT4G04840	Q8GWF4	MSRB6_ARATH Peptide methionine sulfoxide reductase B6	Unique in *pldα1-1*		n.a.		6.01	2	19.61	3
At5g17820	Q43729	PER57_ARATH Peroxidase 57	1.58		0.025		40.87	10	50.16	43
At3g21770	Q9LSY7	PER30_ARATH Peroxidase 30	Unique in *pldα1-1*	Unique in *pldα1-2*	n.a.	n.a.	7.05	2	10.33	3
At2g38380	P24102	PER22_ARATH Peroxidase 22	1.79	1.99	0.031	0.005	13.69	3	18.05	19
At2g18150	Q9SI16	PER15_ARATH Peroxidase 15	Unique in Col-0		n.a.		13.63	4	21.60	6
AT4G21960	Q9SB81	PER42_ARATH Peroxidase 42	0.62		0.001		7.44	2	9.70	3
AT4G35090	P25819	CATA2_ARATH Catalase-2		0.76		0.033	40.79	9	32.32	68
AT1G02920	Q9SRY5	GSTF7_ARATH Glutathione S-transferase F7	0.31	0.25	0.053	0.036	34.78	9	55.02	21
At3g04780	Q9SQZ9	PITH1_ARATH PITH domain-containing protein At3g04780		0.68		0.05	7.05	2	25.00	3
AT5G28840	Q93VR3	GME_ARATH GDP-mannose 3,5-epimerase	0.45		0.013		10.83	3	11.14	7
AT1G66280	Q9C8Y9	BGL22_ARATH Beta-glucosidase 22		1.75		0.026	83.42	19	49.62	129
AT1G66270	Q9C525	BGL21_ARATH Beta-glucosidase 21		1.78		0.020	74.98	18	53.44	115
		**Cytoskeleton**								
AT4G00680	Q570Y6	ADF8_ARATH Actin-depolymerizing factor 8	0.77		0		6.61	2	23.57	3
AT2G37620	P0CJ46	ACT1_ARATH Actin-1	0.68		0.039		22.96	6	26.53	13
AT1G01750	Q9LQ81	ADF10_ARATH Actin-depolymerizing factor 10	0.66		0.015		6.59	2	20.00	3
At3g10220	Q67Z52	TBCB_ARATH Tubulin-folding cofactor B	Unique in Col-0		n.a.		10.23	3	21.81	3
AT3G46010	Q39250	ADF1_ARATH Actin-depolymerizing factor 1		0.24		0.014	7.54	2	23.74	5
At5g49510	P57741	PFD3_ARATH Probable prefoldin subunit 3		Unique in *pldα1-2*		n.a.	8.09	2	19.49	3
		**Cell cycle and cell proliferation**								
At1g07370	Q9M7Q7	PCNA1_ARATH Proliferating cellular nuclear antigen 1	0.47		0.04		13.75	4	26.62	7
AT3G16640	P31265	TCTP1_ARATH Translationally-controlled tumor protein 1	1.49		0.054		26.34	6	41.07	53
		**Membrane transport**								
At3g58730	Q9XGM1	VATD_ARATH V-type proton ATPase subunit D	0.57		0.027		10.27	3	18.01	4
At3g28715	Q9LHA4	VA0D2_ARATH V-type proton ATPase subunit d2	0.20		0.017		14.03	4	21.08	5
At4g32285	Q8S9J8	CAP1_ARATH Probable clathrin assembly protein At4g32285	Unique in Col-0	Unique in Col-0	n.a.	n.a.	9.44	3	6.77	3
At3g01340	Q9SRI1	SC13A_ARATH Protein transport protein SEC13 homolog A	2.55	2.97	0.001	0.011	12.12	3	22.85	5
At3g15950	Q9LSB4	NAI2_ARATH TSA1-like protein	1.33	1.71	0.049	0.003	96.37	22	43.78	131

At2g45140	Q9SHC8	VAP12_ARATH Vesicle-associated protein 1-2	1.51		0.047		7.21	2	14.23	4
At3g56190	Q9SPE6	SNAA2_ARATH Alpha-soluble NSF attachment protein 2	1.65		0.043		19.95	5	25.61	19
At3g47810	Q9STT2	VPS29_ARATH Vacuolar protein sorting-associated protein 29	Unique in *pldα1-1*		n.a.		6.95	2	25.79	4
At5g41950	Q9FHY8	HLB1_ARATH Protein HLB1	0.36		0.001		10.63	3	8.85	4
At3g24350	Q9LK09	SYP32_ARATH Syntaxin-32		Unique in *pldα1-2*		n.a.	7.17	2	10.37	4
At2g20990	Q9SKR2	SYT1_ARATH Synaptotagmin-1		Unique in *pldα1-2*		n.a.	7.13	2	5.36	4
At1g79830	Q0WVL7	GOGC5_ARATH Golgin candidate 5		Unique in *pldα1-2*		n.a.	6.45	2	2.82	5
At5g16760	Q9SBA5	ITPK1_ARATH Inositol-tetrakisphosphate 1-kinase 1		2.38		0.014	13.42	4	17.55	7
At2g24940	Q9SK39	SBP3_ARATH Probable steroid-binding protein 3		1.53		0.055	17.51	4	48.00	32
At1g30690	Q94C59	PATL4_ARATH Patellin-4		2.30		0.028	18.05	4	13.52	13
		**Cell wall**								
AT5G10560	Q9LXA8	BXL6_ARATH Probable beta-D-xylosidase 6		Unique in *pldα1-2*		n.a.	10.06	3	5.18	4
AT1G12780	Q42605	UGE1_ARATH Bifunctional UDP-glucose 4-epimerase and UDP-xylose 4-epimerase 1		Unique in *pldα1-2*		n.a.	7.53	2	11.97	3
AT4G25250	Q9SB38	PMEI4_ARATH Pectinesterase inhibitor 4		0.79		0.001	6.75	2	17.59	3
At4g11190	Q9T017	DIR13_ARATH Dirigent protein 13		2.81		0.03	7.85	2	19.02	5
AT1G03870	Q9ZWA8	FLA9_ARATH Fasciclin-like arabinogalactan protein 9		1.65		0.036	11.89	3	15.79	7
		**Mitochondrial protein import and quality control**								
AT1G72750	Q38820	TI232_ARATH Mitochondrial import inner membrane translocase subunit TIM23-2	0.36			0.009	7.09	2	18.62	4
AT1G61570	Q9XH48	TIM13_ARATH Mitochondrial import inner membrane translocase subunit TIM13		0.55		0.001	7.90	2	40.23	5
At5g45390	Q94B60	CLPP4_ARATH ATP-dependent Clp protease proteolytic subunit 4, chloroplastic		0.31		0.044	8.17	2	18.84	6
At5g23140	Q9FN42	CLPP2_ARATH ATP-dependent Clp protease proteolytic subunit 2, mitochondrial	Unique in *pldα1-1*		n.a.		7.54	2	18.67	3
At2g20530	Q9SIL6	PHB6_ARATH Prohibitin-6, mitochondrial	0.43		0.052		8.94	2	11.89	18
At3g19170	Q9LJL3	PREP1_ARATH Presequence protease 1, chloroplastic/mitochondrial		Unique in *pldα1-2*		n.a.	6.91	2	3.33	3
		**Mitochondrial respiratory chain**								
AT3G54110	O81845	PUMP1_ARATH Mitochondrial uncoupling protein 1	1.67	2.14	0.02	0.001	6.59	2	7.52	3
AT5G11770	Q42577	NDUS7_ARATH NADH dehydrogenase [ubiquinone] iron-sulfur protein 7, mitochondrial		0.78		0.008	3.61	1	6.42	3
AT2G33220	O49313	NDADB_ARATH NADH dehydrogenase [ubiquinone] 1 alpha subcomplex subunit 13-B		0.45		0.044	7.68	2	23.78	8

ATMG00510	P93306	NDUS2_ARATH NADH dehydrogenase [ubiquinone] iron-sulfur protein 2	0.50	0.56	0.028	0.054	11.54	3	11.17	6
AT3G52300	Q9FT52	ATP5H_ARATH ATP synthase subunit d, mitochondrial	1.69	1.57	0.047	0.046	31.83	9	37.50	22
AT5G13450	Q96251	ATPO_ARATH ATP synthase subunit O, mitochondrial	1.53		0.005		27.87	7	42.86	72
AT1G22450	Q9S7L9	CX6B1_ARATH Cytochrome c oxidase subunit 6b-1	Unique in *pldα1-1*	Unique in *pldα1-2*	n.a.	n.a.	8.19	2	17.80	4
AT5G13490	P40941	ADT2_ARATH ADP,ATP carrier protein 2, mitochondrial	0.51		0.012		14.52	4	17.40	16
		**Proteolysis**								
AT5G67360	O65351	SBT17_ARATH Subtilisin-like protease SBT1.7	0.49		0.001		11.21	3	9.38	5
At5g42970	Q8L5U0	CSN4_ARATH COP9 signalosome complex subunit 4	3.25		0.001		10.35	3	10.33	6
At3g59990	Q56Y85	MAP22_ARATH Methionine aminopeptidase 2B	2.30		0.006		24.49	5	19.59	20
At1g20850	Q9LM66	XCP2_ARATH Cysteine protease XCP2		Unique in *pldα1-2*		n.a.	7.74	2	10.11	3
At4g20850	F4JVN6	TPPII_ARATH Tripeptidyl-peptidase 2		1.77		0.001	58.52	15	18.55	41
At2g36060	Q9SJ44	UEV1C_ARATH Ubiquitin-conjugating enzyme E2 variant 1C		2.27		0.026	9.05	2	24.83	13
AT5G23540	Q9LT08	PSDE_ARATH 26S proteasome non-ATPase regulatory subunit 14 homolog	0.76	0.72	0.007	0.038	25.00	6	35.39	40
At5g58290	Q9SEI4	PRS6B_ARATH 26S proteasome regulatory subunit 6B homolog		1.75		0.032	35.33	8	37.50	40
AT5G09900	Q9FIB6	PS12A_ARATH 26S proteasome non-ATPase regulatory subunit 12 homolog A		0.40		0.041	7.24	2	8.82	3
AT5G20000	Q94BQ2	PRS8B_ARATH 26S proteasome regulatory subunit 8 homolog B		1.84		0.034	29.23	7	23.39	31
At5g35590	O81146	PSA6A_ARATH Proteasome subunit alpha type-6-A		2.33		0.038	18.83	5	27.24	18
At2g39960	P58684	SPCS2_ARATH Probable signal peptidase complex subunit 2		0.58		0.039	9.50	3	18.75	3
		**Protein folding**								
AT5G60640	Q9FF55	PDI14_ARATH Protein disulfide isomerase-like 1-4	1.84		0.025		19.04	5	13.40	12
AT5G58710	Q9SP02	CP20A_ARATH Peptidyl-prolyl cis-trans isomerase CYP20-1	1.13	1.56	0.004	0.013	7.75	2	16.67	4
AT2G04030	Q9SIF2	HS905_ARATH Heat shock protein 90-5, chloroplastic		0.39		0.031	36.71	9	22.69	28
		**Translation and protein synthesis**								
At3g15000	Q9LKA5	MORF8_ARATH Multiple organellar RNA editing factor 8, chloroplastic/mitochondrial	0.79		0.001		6.60	2	6.08	3
At5g60790	Q9FJH6	AB1F_ARATH ABC transporter F family member 1	Unique in Col-0	0.38	n.a.	0.03	6.98	2	6.22	3
AT5G61030	Q9FNR1	RBG3_ARATH Glycine-rich RNA-binding protein 3, mitochondrial	0.37		0.04		7.15	2	12.30	4
AT5G23740	P42733	RS113_ARATH 40S ribosomal protein S11-3		0.16		0.03	10.03	3	25.79	3

AT5G18380	Q42340	RS163_ARATH 40S ribosomal protein S16-3	Unique in Col-0			n.a.	10.69	3	18.49	6
AT2G09990	Q9SK22	RS161_ARATH 40S ribosomal protein S16-1	Unique in Col-0			n.a.	6.49	2	17.12	3
AT5G61170	Q9FNP8	RS193_ARATH 40S ribosomal protein S19-3		1.80		0.045	16.50	4	30.07	25
AT5G46430	Q9FHG2	RL322_ARATH 60S ribosomal protein L32-2	0.48			0.021	7.49	2	21.05	3
AT5G22440	P59231	R10A3_ARATH 60S ribosomal protein L10a-3		1.69		0.046	22.23	6	28.57	25
AT4G00810	O23095	RLA12_ARATH 60S acidic ribosomal protein P1-2	1.52		0.052		9.86	2	50.44	18
At2g39460	Q8LD46	R23A1_ARATH 60S ribosomal protein L23a-1	20.82	7.62	0.001	0.012	9.56	3	24.68	11
At3g57290	Q9C5Z3	EIF3E_ARATH Eukaryotic translation initiation factor 3 subunit E	0.59		0.001		12.24	3	10.88	9
At3g51800	Q96327	EBP1_ARATH ERBB-3 BINDING PROTEIN 1	1.79		0.032		24.68	6	28.06	21
AT4G17520	O23593	RGGB_ARATH RGG repeats nuclear RNA binding protein B	1.67		0.032		8.36	2	11.11	6
AT1G14610	P93736	SYVM1_ARATH Valine--tRNA ligase, mitochondrial 1	0.76		0.003		7.44	2	4.51	3
AT5G26830	O04630	SYTM1_ARATH Threonine--tRNA ligase, mitochondrial 1		0.49		0.041	14.08	4	7.19	6
At3g02760	F4IYF8	SYHC_ARATH Histidine--tRNA ligase, cytoplasmic		0.42		0.043	10.89	3	6.80	4
AT5G11170	Q56XG6	RH15_ARATH DEAD-box ATP-dependent RNA helicase 15		0.55		0.05	25.39	6	22.95	30
At1g73230	Q9CAT7	BTF3L_ARATH Nascent polypeptide-associated complex subunit beta		0.58		0.033	23.69	6	41.82	17
At2g33840	Q8S9J2	BTF3L_ARATH Nascent polypeptide-associated complex subunit beta		0.71		0.049	8.37	2	7.01	6
AT3G12390	Q9LHG9	NACA1_ARATH Nascent polypeptide-associated complex subunit alpha-like protein 1	1.82	1.91	0.052	0.029	33.15	7	47.78	56
At5g13850	Q6ICZ8	NACA3_ARATH Nascent polypeptide-associated complex subunit alpha-like protein 3		2.28		0.051	17.89	4	39.22	22
		**Nuclear import**								
AT1G02690	Q9FWY7	IMPA6_ARATH Importin subunit alpha-6	0.40		0.049		7.31	2	5.39	5
AT3G63130	Q9LE82	RAGP1_ARATH RAN GTPase-activating protein 1		Unique in *pldα1-2*		n.a.	6.57	2	5.61	3
		**Glucosinolate biosynthesis**								
AT1G31180	Q9SA14	LEU31_ARATH 3-isopropylmalate dehydrogenase 1, chloroplastic	1.52	1.55	0.019	0.02	27.76	7	17.33	45
AT1G80560	P93832	LEU32_ARATH 3-isopropylmalate dehydrogenase 2, chloroplastic		2.33		0.055	20.95	5	17.04	30
AT5G14200	Q9FMT1	LEU33_ARATH 3-isopropylmalate dehydrogenase 3, chloroplastic	1.49	1.61	0.002	0.001	46.68	11	35.21	90
AT2G43090	Q9ZW85	LEUD3_ARATH 3-isopropylmalate dehydratase small subunit 3		1.60		0.044	17.44	4	24.70	36

AT5G23010	Q9FG67	MAM1_ARATH Methylthioalkylmalate synthase 1, chloroplastic	1.13	1.74	0.001	0.036	6.79	2	6.13	4
AT4G31500	O65782	C83B1_ARATH Cytochrome P450 83B1	4.06		0.001		6.51	2	8.02	3
AT2G30860	O80852	GSTF9_ARATH Glutathione S-transferase F9	1.55		0.035		42.01	9	48.37	93
AT1G21100	Q9LPU5	IGMT1_ARATH Indole glucosinolate O-methyltransferase 1		0.66		0.001	6.99	2	6.43	4
AT2G14750	Q43295	APK1_ARATH Adenylyl-sulfate kinase 1, chloroplastic		Unique in *pldα1-2*		n.a.	10.00	3	21.01	3
AT5G27380	P46416	GSHB_ARATH Glutathione synthetase, chloroplastic		Unique in Col-0		n.a.	6.56	2	6.12	3
		**Camalexin biosynthesis**								
AT1G02930	P42760	GSTF6_ARATH Glutathione S-transferase F6	0.29	0.26	0.049	0.029	24.60	6	47.12	18
At2g30750	O49340	C71AC_ARATH Cytochrome P450 71A12	Unique in Col-0	Unique in Col-0	n.a.	n.a.	14.29	4	15.29	5
		**Auxin biosynthesis and transport**								
AT3G44310	P32961	NRL1_ARATH Nitrilase 1	1.79	1.79	0.018	0.023	13.40	3	16.76	22
AT3G02260	Q9SRU2	BIG_ARATH Auxin transport protein BIG	1.97		0.009		7.52	2	0.84	3
AT5G54810	P14671	TRPB1_ARATH Tryptophan synthase beta chain 1, chloroplastic	0.48		0.051		25.05	6	21.06	27
		**Fatty acid metabolism**								
At5g64440	Q7XJJ7	FAAH_ARATH Fatty acid amide hydrolase		Unique in *pldα1-2*		n.a.	7.85	2	5.27	3
At1g54000	Q1H583	GDL18_ARATH GDSL esterase/lipase 22		1.72		0.006	50.51	11	46.29	110
At1g54010	Q8W4H8	GDL19_ARATH Inactive GDSL esterase/lipase-like protein 23		1.59		0.054	45.38	11	44.04	79
		**Metabolism**								
AT1G31860	O82768	HIS2_ARATH Histidine biosynthesis bifunctional protein hisIE, chloroplastic		Unique in *pldα1-2*		n.a.	8.78	2	11.74	4
AT1G60680	Q84M96	ALKR2_ARATH Probable aldo-keto reductase 2		0.41		0.000	8.40	2	7.80	4
AT2G43940	Q6AWU6	HOL3_ARATH Probable thiol methyltransferase 2		1.79		0.000	7.19	2	18.58	6
AT5G17310	P57751	UGPA1_ARATH UTP--glucose-1-phosphate uridylyltransferase 1		1.54		0.038	43.14	11	35.53	50
AT5G51970	Q9FJ95	DHSO_ARATH Sorbitol dehydrogenase		2.77		0.005	17.30	4	21.98	20
AT3G57050	P53780	METC_ARATH Cystathionine beta-lyase, chloroplastic		5.50		0.005	8.45	2	7.97	7
AT5G13110	Q9FY99	G6PD2_ARATH Glucose-6-phosphate 1-dehydrogenase 2, chloroplastic		2.58		0.007	19.33	5	12.08	16
AT5G53460	Q9LV03	GLUT1_ARATH Glutamate synthase 1 [NADH], chloroplastic		0.41		0.009	10.62	3	2.72	3
AT5G57440	Q8VZP1	GPP2_ARATH (DL)-glycerol-3-phosphatase 2		0.36		0.011	10.95	3	22.08	5
AT1G23800	Q8S528	AL2B7_ARATH Aldehyde dehydrogenase family 2 member B7, mitochondrial		2.51		0.032	19.14	5	18.54	18

AT4G26900	Q9SZ30	HIS4_ARATH Imidazole glycerol phosphate synthase hisHF, chloroplastic		1.82		0.035	12.26	3	8.61	9
AT2G45790	O80840	PMM_ARATH Phosphomannomutase		1.92		0.037	26.59	7	37.40	20
AT3G48560	P17597	ILVB_ARATH Acetolactate synthase, chloroplastic		0.35		0.043	16.00	4	11.94	5
AT2G26080	O80988	GCSP2_ARATH Glycine dehydrogenase (decarboxylating) 2, mitochondrial		0.63		0.048	24.24	6	11.21	18
AT3G15730	Q38882	PLDA1_ARATH Phospholipase D alpha 1	Unique in Col-0	Unique in Col-0	n.a.	n.a.	25.95	6	15.31	15
AT5G66120	Q8VYV7	DHQS_ARATH 3-dehydroquinate synthase, chloroplastic	0.39	0.44	0.046	0.01	13.45	4	15.38	6
AT1G79500	Q9AV97	KDSA1_ARATH 2-dehydro-3-deoxyphosphooctonate aldolase 1	Unique in *pldα1-1*	Unique in *pldα1-2*	n.a.	n.a.	7.54	2	9.66	4
AT5G43280	Q9FHR8	DCI1_ARATH Delta(3,5)-Delta(2,4)-dienoyl-CoA isomerase, peroxisomal	0.44	0.3	0.011	0.003	11.95	3	20.14	7
AT5G47720	Q9FIK7	THIC2_ARATH Probable acetyl-CoA acetyltransferase, cytosolic 2	0.59	1.71	0.042	0.055	11.47	3	14.94	4
AT5G55070	Q9FLQ4	ODO2A_ARATH Dihydrolipoyllysine-residue succinyltransferase component of 2-oxoglutarate dehydrogenase complex 1, mitochondrial	8.28	5.54	0.008	0.037	8.26	2	8.62	10
AT2G13560	Q9SIU0	MAO1_ARATH NAD-dependent malic enzyme 1, mitochondrial	4.99	2.1	0.011	0.009	9.70	3	9.63	4
AT5G65720	O49543	MNIF1_ARATH Cysteine desulfurase, mitochondrial	Unique in *pldα1-1*		n.a.		10.59	3	11.48	3
AT5G66280	Q9SNY3	GMD1_ARATH GDP-mannose 4,6 dehydratase 1	Unique in *pldα1-1*		n.a.		8.08	2	13.30	4
AT2G18230	P21216	IPYR2_ARATH Soluble inorganic pyrophosphatase 2	Unique in *pldα1-1*		n.a.		6.93	2	20.64	3
AT2G44530	O64888	KPRS5_ARATH Ribose-phosphate pyrophosphokinase 5, chloroplastic	Unique in *pldα1-1*		n.a.		6.92	2	11.42	3
AT4G26910	Q8H107	ODO2B_ARATH Dihydrolipoyllysine-residue succinyltransferase component of 2-oxoglutarate dehydrogenase complex 2, mitochondrial	12.21		0.001		8.09	2	8.62	4
AT5G01410	Q8L940	PDX13_ARATH Pyridoxal 5'-phosphate synthase subunit PDX1.3	0.45		0.001		7.03	2	9.06	3
AT5G20250	Q8RX87	RFS6_ARATH Probable galactinol--sucrose galactosyltransferase 6	1.95		0.001		6.93	2	3.60	3
ATCG00120	P56757	ATPA_ARATH ATP synthase subunit alpha, chloroplastic	6.54		0.001		6.56	2	6.51	6
AT1G63000	Q9LQ04	RMLCD_ARATH Bifunctional dTDP-4-dehydrorhamnose 3,5-epimerase/dTDP-4-dehydrorhamnose reductase	1.38	1.6	0.038	0.029	24.68	6	33.55	43
AT4G22570	Q9SUW2	APT3_ARATH Adenine phosphoribosyltransferase 3	0.37		0.009		6.60	2	18.03	4

AT1G17745	O04130	SERA2_ARATH D-3-phosphoglycerate dehydrogenase 2, chloroplastic	0.30		0.021		18.10	5	11.54	7
AT2G47510	P93033	FUM1_ARATH Fumarate hydratase 1, mitochondrial	1.57		0.040		25.70	6	25.00	24
AT4G22930	O04904	PYRC_ARATH Dihydroorotase, mitochondrial	2.77		0.044		19.59	5	20.69	15
AT4G09510	Q67XD9	CINV2_ARATH Alkaline/neutral invertase CINV2	0.46		0.044		7.84	2	8.60	8
AT4G25840	F4JTE7	GPP1_ARATH (DL)-glycerol-3-phosphatase 1, mitochondrial	2.83		0.054		9.66	3	18.46	3
AT1G13280	Q93ZC5	AOC4_ARATH Allene oxide cyclase 4, chloroplastic	1.60		0.055		7.72	2	14.96	7
AT1G16350	Q9SA34	IMDH2_ARATH Inosine-5'-monophosphate dehydrogenase 2		2.39		0.043	20.65	5	16.53	15
AT1G67280	Q8W593	LGUC_ARATH Probable lactoylglutathione lyase, chloroplastic		2.75		0.021	7.74	2	14.00	6
AT3G08590	Q9M9K1	PMG2_ARATH Probable 2,3-bisphosphoglycerate-independent phosphoglycerate mutase 2		0.75		0.053	69.89	17	49.46	86
AT4G34215	Q8L9J9	CAES_ARATH Probable carbohydrate esterase At4g34215		0.74		0.031	3.69	1	5.77	3
AT4G33010	Q94B78	GCSP1_ARATH Glycine dehydrogenase (decarboxylating) 1, mitochondrial		0.67		0.033	23.02	6	9.84	21
AT3G48170	Q9STS1	BADH2_ARATH Betaine aldehyde dehydrogenase 2, mitochondrial	4.26			0.005	7.97	2	8.15	5
AT5G20960	Q7G193	ALDO1_ARATH Indole-3-acetaldehyde oxidase		0.64		0.000	10.19	3	4.17	3
AT2G37040	P35510	PAL1_ARATH Phenylalanine ammonia-lyase 1		0.73		0.052	33.72	8	19.17	41
		**Unknown**								
AT4G28440	O49453	Y4844_ARATH Uncharacterized protein At4g28440		0.80		0.002	4.10	1	10.46	3
		**Histones**								
At1g52740	Q9C944	H2AV3_ARATH Probable histone H2A variant 3		Unique in *pldα1-2*		n.a.	9.23	2	32.09	4
		**Methylation**								
At3g12290	Q9LHH7	FOLD2_ARATH Bifunctional protein FolD 2	0.53		0.023		11.63	3	16.05	12
At1g15340	Q9XI36	MBD10_ARATH Methyl-CpG-binding domain-containing protein 10		Unique in *pldα1-2*		n.a.	11.96	3	12.50	5

Table S2. Distribution of protein families among the differentially abundant proteins found in roots of *pldα1-1* and *pdα1-2* mutants.

IPS Family	#Seqs	Accession	Protein name	Fold change	
				pldα1-1 vs Col-0	*pldα1-2* vs Col-0
(IPR036291) NAD(P)-binding domain superfamily	9	O04130	SERA2_ARATH D-3-phosphoglycerate dehydrogenase 2, chloroplastic	0.30	
		Q93VR3	GME_ARATH GDP-mannose 3,5-epimerase	0.45	
		Q9LHH7	FOLD2_ARATH Bifunctional protein FolD 2	0.53	
		Q9LQ04	RMLCD_ARATH Bifunctional dTDP-4-dehydrorhamnose 3,5-epimerase/dTDP-4-dehydrorhamnose reductase	1.38	
		Q9SIU0	MAO1_ARATH NAD-dependent malic enzyme 1, mitochondrial	4.99	1.60
		Q9SNY3	GMD1_ARATH GDP-mannose 4,6 dehydratase 1	Unique in *pldα1-1*	2.10
		Q9FY99	G6PD2_ARATH Glucose-6-phosphate 1-dehydrogenase 2, chloroplastic		
		Q9FJ95	DHSO_ARATH Sorbitol dehydrogenase		2.58
		Q42605	UGE1_ARATH Bifunctional UDP-glucose 4-epimerase and UDP-xylose 4-epimerase 1		2.77
(IPR036390) Winged helix DNA-binding domain superfamily	7	Q9C5Z3	EIF3E_ARATH Eukaryotic translation initiation factor 3 subunit E	0.59	
		Q96327	EBP1_ARATH ERBB-3 BINDING PROTEIN 1	1.79	
		Q56Y85	MAP22_ARATH Methionine aminopeptidase 2B	2.30	
		Q8L5U0	CSN4_ARATH COP9 signalosome complex subunit 4	3.25	
		Q9FIB6	PS12A_ARATH 26S proteasome non-ATPase regulatory subunit 12 homolog A		0.40
		Q9LPU5	IGMT1_ARATH Indole glucosinolate O-methyltransferase 1		0.66
		Q9FNP8	RS193_ARATH 40S ribosomal protein S19-3		1.80
(IPR010255) Haem peroxidase	6	Q9SB81	PER42_ARATH Peroxidase 42	0.62	
		Q43729	PER57_ARATH Peroxidase 57	1.58	
		P24102	PER22_ARATH Peroxidase 22	1.79	1.99
		Q42592	APXS_ARATH L-ascorbate peroxidase S, chloroplastic/mitochondrial	2.25	

		Q9LSY7	PER30_ARATH Peroxidase 30	Unique in *pldα1-1*	Unique in *pldα1-2*
		Q9SI16	PER15_ARATH Peroxidase 15	Unique in Col-0	
(IPR013785) Aldolase-type TIM barrel	6	Q8L940	PDX13_ARATH Pyridoxal 5'-phosphate synthase subunit PDX1.3	0.45	
		Q9FG67	MAM1_ARATH Methylthioalkylmalate synthase 1, chloroplastic	1.13	1.74
		Q8RX87	RFS6_ARATH Probable galactinol--sucrose galactosyltransferase 6	1.95	
		Q9AV97	KDSA1_ARATH 2-dehydro-3-deoxyphosphooctonate aldolase 1	Unique in *pldα1-1*	Unique in *pldα1-2*
		Q9LV03	GLUT1_ARATH Glutamate synthase 1 [NADH], chloroplastic		0.41
		Q9SZ30	HIS4_ARATH Imidazole glycerol phosphate synthase hisHF, chloroplastic		1.82
(IPR027417) P-loop containing nucleoside triphosphate hydrolase	6	P56757	ATPA_ARATH ATP synthase subunit alpha, chloroplastic	6.54	
		Q9FJH6	AB1F_ARATH ABC transporter F family member 1	Unique in Col-0	0.38
		Q56XG6	RH15_ARATH DEAD-box ATP-dependent RNA helicase 15		0.55
		Q9SEI4	PRS6B_ARATH 26S proteasome regulatory subunit 6B homolog		1.75
		Q94BQ2	PRS8B_ARATH 26S proteasome regulatory subunit 8 homolog B		1.84
		Q43295	APK1_ARATH Adenylyl-sulfate kinase 1, chloroplastic		Unique in *pldα1-2*
(IPR000823) Plant peroxidase	5	Q9SB81	PER42_ARATH Peroxidase 42	0.62	
		Q43729	PER57_ARATH Peroxidase 57	1.58	
		P24102	PER22_ARATH Peroxidase 22	1.79	1.99
		Q9LSY7	PER30_ARATH Peroxidase 30	Unique in *pldα1-1*	Unique in *pldα1-2*
		Q9SI16	PER15_ARATH Peroxidase 15	Unique in Col-0	
(IPR017853) Glycoside hydrolase superfamily	5	Q8RX87	RFS6_ARATH Probable galactinol--sucrose galactosyltransferase 6	1.95	
		Q8VZJ2	BGNEM_ARATH Probable glucan endo-1,3-beta-glucosidase At4g16260		0.59
		Q9C8Y9	BGL22_ARATH Beta-glucosidase 22		1.75
		Q9C525	BGL21_ARATH Beta-glucosidase 21		1.78

		Q9LXA8	BXL6_ARATH Probable beta-D-xylosidase 6		Unique in *pldα1-2*
(IPR036388) Winged helix-like DNA-binding domain superfamily	5	Q96327	EBP1_ARATH ERBB-3 BINDING PROTEIN 1	1.79	
		Q56Y85	MAP22_ARATH Methionine aminopeptidase 2B	2.30	
		Q8L5U0	CSN4_ARATH COP9 signalosome complex subunit 4	3.25	
		Q9FIB6	PS12A_ARATH 26S proteasome non-ATPase regulatory subunit 12 homolog A		0.40
		Q9LPU5	IGMT1_ARATH Indole glucosinolate O-methyltransferase 1		0.66
(IPR015421) Pyridoxal phosphate-dependent transferase, major domain	4	O49543	MNIF1_ARATH Cysteine desulfurase, mitochondrial	Unique in *pldα1-1*	
		O80988	GCSP2_ARATH Glycine dehydrogenase (decarboxylating) 2, mitochondrial		0.63
		P53780	METC_ARATH Cystathionine beta-lyase, chloroplastic		5.50
		Q94B78	GCSP1_ARATH Glycine dehydrogenase (decarboxylating) 1, mitochondrial		0.67
(IPR036249) Thioredoxin-like superfamily	4	P42760	GSTF6_ARATH Glutathione S-transferase F6	0.29	0.26
		Q9SRY5	GSTF7_ARATH Glutathione S-transferase F7	0.31	0.25
		O80852	GSTF9_ARATH Glutathione S-transferase F9	1.55	
		Q9FF55	PDI14_ARATH Protein disulfide isomerase-like 1-4	1.84	
(IPR015422) Pyridoxal phosphate-dependent transferase domain 1	4	O49543	MNIF1_ARATH Cysteine desulfurase, mitochondrial	Unique in *pldα1-1*	
		O80988	GCSP2_ARATH Glycine dehydrogenase (decarboxylating) 2, mitochondrial		0.63
		P53780	METC_ARATH Cystathionine beta-lyase, chloroplastic		5.50
		Q94B78	GCSP1_ARATH Glycine dehydrogenase (decarboxylating) 1, mitochondrial		0.67
(IPR015424) Pyridoxal phosphate-dependent transferase	4	O49543	MNIF1_ARATH Cysteine desulfurase, mitochondrial	Unique in *pldα1-1*	
		O80988	GCSP2_ARATH Glycine dehydrogenase (decarboxylating) 2, mitochondrial		0.63
		P53780	METC_ARATH Cystathionine beta-lyase, chloroplastic		5.50
		Q94B78	GCSP1_ARATH Glycine dehydrogenase (decarboxylating) 1, mitochondrial		0.67

(IPR017904) ADF/Cofilin	3	Q9LQ81	ADF10_ARATH Actin-depolymerizing factor 10	0.66	
		Q570Y6	ADF8_ARATH Actin-depolymerizing factor 8	0.77	
		Q39250	ADF1_ARATH Actin-depolymerizing factor 1		0.24
(IPR040079) Glutathione Transferase family	3	P42760	GSTF6_ARATH Glutathione S-transferase F6	0.29	0.26
		Q9SRY5	GSTF7_ARATH Glutathione S-transferase F7	0.31	0.25
		O80852	GSTF9_ARATH Glutathione S-transferase F9	1.55	
(IPR038187) NAC A/B domain superfamily	3	Q9LHG9	NACA1_ARATH Nascent polypeptide-associated complex subunit alpha-like protein 1	1.82	1.91
		Q9CAT7	BTF3L_ARATH Nascent polypeptide-associated complex subunit beta		0.58
		Q6ICZ8	NACA3_ARATH Nascent polypeptide-associated complex subunit alpha-like protein 3		2.28
(IPR004429) Isopropylmalate dehydrogenase	3	Q9FMT1	LEU33_ARATH 3-isopropylmalate dehydrogenase 3, chloroplastic	1.49	1.61
		Q9SA14	LEU31_ARATH 3-isopropylmalate dehydrogenase 1, chloroplastic	1.52	1.55
		P93832	LEU32_ARATH 3-isopropylmalate dehydrogenase 2, chloroplastic		2.33
(IPR029045) ClpP/crotonase-like domain superfamily	3	Q9FHR8	DCI1_ARATH Delta(3,5)-Delta(2,4)-dienoyl-CoA isomerase, peroxisomal	0.44	0.30
		Q9FN42	CLPP2_ARATH ATP-dependent Clp protease proteolytic subunit 2, mitochondrial	Unique in *pldα1-1*	
		Q94B60	CLPP4_ARATH ATP-dependent Clp protease proteolytic subunit 4, chloroplastic		0.31
(IPR011990) Tetratricopeptide-like helical domain superfamily	3	Q9FHY8	HLB1_ARATH Protein HLB1	0.36	
		Q9SPE6	SNAA2_ARATH Alpha-soluble NSF attachment protein 2	1.65	
		Q9LYW9	DNJ36_ARATH DnaJ protein P58IPK homolog	4.03	4.11
(IPR020568) Ribosomal protein S5 domain 2-type fold	3	Q9SK22	RS161_ARATH 40S ribosomal protein S16-1	Unique in Col-0	
		Q42340	RS163_ARATH 40S ribosomal protein S16-3	Unique in Col-0	
		Q9SIF2	HS905_ARATH Heat shock protein 90-5, chloroplastic		0.39
(IPR035892) C2 domain superfamily	3	Q38882	PLDA1_ARATH Phospholipase D alpha 1	Unique in Col-0	Unique in Col-0

		Q9ZVF1	CAR10_ARATH Protein C2-DOMAIN ABA-RELATED 10	Unique in Col-0	
		Q9SKR2	SYT1_ARATH Synaptotagmin-1		Unique in *pldα1-2*
(IPR036282) Glutathione S-transferase, C-terminal domain superfamily	3	P42760	GSTF6_ARATH Glutathione S-transferase F6	0.29	0.26
		Q9SRY5	GSTF7_ARATH Glutathione S-transferase F7	0.31	0.25
		O80852	GSTF9_ARATH Glutathione S-transferase F9	1.55	
(IPR029006) ADF-H/Gelsolin-like domain superfamily	3	Q9LQ81	ADF10_ARATH Actin-depolymerizing factor 10	0.66	
		Q570Y6	ADF8_ARATH Actin-depolymerizing factor 8	0.77	
		Q39250	ADF1_ARATH Actin-depolymerizing factor 1		0.24
(IPR023214) HAD superfamily	3	F4JTE7	GPP1_ARATH (DL)-glycerol-3-phosphatase 1, mitochondrial	2.83	
		Q8VZP1	GPP2_ARATH (DL)-glycerol-3-phosphatase 2		0.36
		O80840	PMM_ARATH Phosphomannomutase		1.92
(IPR036412) HAD-like superfamily	3	F4JTE7	GPP1_ARATH (DL)-glycerol-3-phosphatase 1, mitochondrial	2.83	
		Q8VZP1	GPP2_ARATH (DL)-glycerol-3-phosphatase 2		0.36
		O80840	PMM_ARATH Phosphomannomutase		1.92
(IPR036514) SGNH hydrolase superfamily	3	Q8L9J9	CAES_ARATH Probable carbohydrate esterase At4g34215		0.74
		Q8W4H8	GDL19_ARATH Inactive GDSL esterase/lipase-like protein 23		1.59
		Q1H583	GDL18_ARATH GDSL esterase/lipase 22		1.72

Table S3. Gene onthology annotation of differentially abundant proteins found in roots of *pldα1-1* and *pldα1-2* mutants according to biological process in 4th level of gene onthology.

GO ID	GO Name	Parent from 3rd level of onthology (GO accession)	Parents (Name)	Nodescore	# Proteins	Protein Names
GO:0044249	cellular biosynthetic process	GO:0009058, GO:0044237	biosynthetic process, cellular metabolic process	55.91	87	JAL28_ARATH Nitrile-specifier protein 1<br>PCNA1_ARATH Proliferating cellular nuclear antigen 1<br>UGE1_ARATH Bifunctional UDP-glucose 4-epimerase and UDP-xylose 4-epimerase 1<br>DIR13_ARATH Dirigent protein 13<br>PREP1_ARATH Presequence protease 1, chloroplastic/mitochondrial<br>ATP5H_ARATH ATP synthase subunit d, mitochondrial<br>ATPO_ARATH ATP synthase subunit O, mitochondrial<br>RS113_ARATH 40S ribosomal protein S11-3<br>RS163_ARATH 40S ribosomal protein S16-3<br>RS161_ARATH 40S ribosomal protein S16-1<br>RS193_ARATH 40S ribosomal protein S19-3<br>RL322_ARATH 60S ribosomal protein L32-2<br>R10A3_ARATH 60S ribosomal protein L10a-3<br>RLA12_ARATH 60S acidic ribosomal protein P1-2<br>R23A1_ARATH 60S ribosomal protein L23a-1<br>EIF3E_ARATH Eukaryotic translation initiation factor 3 subunit E<br>SYTM1_ARATH Threonine--tRNA ligase, mitochondrial 1<br>SYHC_ARATH Histidine--tRNA ligase, cytoplasmic<br>BTF3L_ARATH Nascent polypeptide-associated complex subunit beta<br>LEU31_ARATH 3-isopropylmalate dehydrogenase 1, chloroplastic<br>LEU32_ARATH 3-isopropylmalate dehydrogenase 2, chloroplastic<br>LEU33_ARATH 3-isopropylmalate dehydrogenase 3, chloroplastic<br>LEUD3_ARATH 3-isopropylmalate dehydratase small subunit 3<br>MAM1_ARATH Methylthioalkylmalate synthase 1, chloroplastic<br>C83B1_ARATH Cytochrome P450 83B1<br>IGMT1_ARATH Indole glucosinolate O-methyltransferase 1<br>APK1_ARATH Adenylyl-sulfate kinase 1, chloroplastic<br>GSHB_ARATH Glutathione synthetase, chloroplastic<br>NRL1_ARATH Nitrilase 1<br>TRPB1_ARATH Tryptophan synthase beta chain 1, chloroplastic<br>HIS2_ARATH Histidine biosynthesis bifunctional protein hisIE, chloroplastic<br>GLUT1_ARATH Glutamate synthase 1 [NADH], chloroplastic<br>GPP2_ARATH (DL)-glycerol-3-phosphatase 2<br>HIS4_ARATH Imidazole glycerol phosphate synthase hisHF, chloroplastic<br>PMM_ARATH Phosphomannomutase<br>ILVB_ARATH Acetolactate synthase, chloroplastic

						DHQS_ARATH 3-dehydroquinate synthase, chloroplastic<br>KDSA1_ARATH 2-dehydro-3-deoxyphosphooctonate aldolase 1<br>MNIF1_ARATH Cysteine desulfurase, mitochondrial<br>GMD1_ARATH GDP-mannose 4,6 dehydratase 1<br>KPRS5_ARATH Ribose-phosphate pyrophosphokinase 5, chloroplastic<br>PDX13_ARATH Pyridoxal 5'-phosphate synthase subunit PDX1.3<br>ATPA_ARATH ATP synthase subunit alpha, chloroplastic<br>RMLCD_ARATH Bifunctional dTDP-4-dehydrorhamnose 3,5-epimerase/dTDP-4-dehydrorhamnose reductase<br>APT3_ARATH Adenine phosphoribosyltransferase 3<br>SERA2_ARATH D-3-phosphoglycerate dehydrogenase 2, chloroplastic<br>PYRC_ARATH Dihydroorotase, mitochondrial<br>GPP1_ARATH (DL)-glycerol-3-phosphatase 1, mitochondrial<br>AOC4_ARATH Allene oxide cyclase 4, chloroplastic<br>IMDH2_ARATH Inosine-5'-monophosphate dehydrogenase 2<br>H2AV3_ARATH Probable histone H2A variant 3<br>FOLD2_ARATH Bifunctional protein FolD 2<br>MBD10_ARATH Methyl-CpG-binding domain-containing protein

GO:1901564	organonitrogen compound metabolic process	GO:0006807, GO:0071704	nitrogen compound metabolic process, organic substance metabolic process	53.72	86	SOX_ARATH Probable sarcosine oxidase<br>JAL28_ARATH Nitrile-specifier protein 1<br>GSTF7_ARATH Glutathione S-transferase F7<br>ATP5H_ARATH ATP synthase subunit d, mitochondrial<br>ATPO_ARATH ATP synthase subunit O, mitochondrial<br>RS113_ARATH 40S ribosomal protein S11-3<br>RS163_ARATH 40S ribosomal protein S16-3<br>RS161_ARATH 40S ribosomal protein S16-1<br>RS193_ARATH 40S ribosomal protein S19-3<br>RL322_ARATH 60S ribosomal protein L32-2<br>R10A3_ARATH 60S ribosomal protein L10a-3<br>RLA12_ARATH 60S acidic ribosomal protein P1-2<br>R23A1_ARATH 60S ribosomal protein L23a-1<br>EIF3E_ARATH Eukaryotic translation initiation factor 3 subunit E<br>SYTM1_ARATH Threonine--tRNA ligase, mitochondrial 1<br>SYHC_ARATH Histidine--tRNA ligase, cytoplasmic<br>LEU31_ARATH 3-isopropylmalate dehydrogenase 1, chloroplastic<br>LEU32_ARATH 3-isopropylmalate dehydrogenase 2, chloroplastic<br>LEU33_ARATH 3-isopropylmalate dehydrogenase 3, chloroplastic<br>LEUD3_ARATH 3-isopropylmalate dehydratase small subunit 3<br>MAM1_ARATH Methylthioalkylmalate synthase 1, chloroplastic<br>C83B1_ARATH Cytochrome P450 83B1<br>GSTF9_ARATH Glutathione S-transferase F9<br>IGMT1_ARATH Indole glucosinolate O-methyltransferase 1<br>APK1_ARATH Adenylyl-sulfate kinase 1, chloroplastic<br>GSHB_ARATH Glutathione synthetase, chloroplastic<br>GSTF6_ARATH Glutathione S-transferase F6<br>NRL1_ARATH Nitrilase 1<br>TRPB1_ARATH Tryptophan synthase beta chain 1, chloroplastic<br>FAAH_ARATH Fatty acid amide hydrolase<br>BGL22_ARATH Beta-glucosidase 22<br>BGL21_ARATH Beta-glucosidase 21<br>HIS2_ARATH Histidine biosynthesis bifunctional protein hisIE, chloroplastic<br>G6PD2_ARATH Glucose-6-phosphate 1-dehydrogenase 2, chloroplastic<br>GLUT1_ARATH Glutamate synthase 1 [NADH], chloroplastic<br>HIS4_ARATH Imidazole glycerol phosphate synthase hisHF, chloroplastic<br>ILVB_ARATH Acetolactate synthase, chloroplastic<br>GCSP2_ARATH Glycine dehydrogenase (decarboxylating) 2, mitochondrial<br>PLDA1_ARATH Phospholipase D alpha 1<br>ODO2A_ARATH Dihydrolipoyllysine-residue succinyltransferase component of 2-oxoglutarate dehydrogenase complex 1, mitochondrial<br>KPRS5_ARATH Ribose-phosphate pyrophosphokinase 5, chloroplastic<br>ODO2B_ARATH Dihydrolipoyllysine-residue succinyltransferase component of 2-oxoglutarate dehydrogenase complex 2, mitochondrial

						PDX13_ARATH Pyridoxal 5'-phosphate synthase subunit PDX1.3<br>ATPA_ARATH ATP synthase subunit alpha, chloroplastic<br>APT3_ARATH Adenine phosphoribosyltransferase 3<br>SERA2_ARATH D-3-phosphoglycerate dehydrogenase 2, chloroplastic<br>PYRC_ARATH Dihydroorotase, mitochondrial<br>IMDH2_ARATH Inosine-5'-monophosphate dehydrogenase 2<br>GCSP1_ARATH Glycine dehydrogenase (decarboxylating) 1, mitochondrial<br>FOLD2_ARATH Bifunctional protein FolD 2

GO:1901576	organic substance biosynthetic process	GO:0009058, GO:0071704	biosynthetic process, organic substance metabolic process	30.33	85	JAL28_ARATH Nitrile-specifier protein 1<br>PCNA1_ARATH Proliferating cellular nuclear antigen 1<br>ITPK1_ARATH Inositol-tetrakisphosphate 1-kinase 1<br>UGE1_ARATH Bifunctional UDP-glucose 4-epimerase and UDP-xylose 4-epimerase 1<br>DIR13_ARATH Dirigent protein 13<br>PREP1_ARATH Presequence protease 1, chloroplastic/mitochondrial<br>ATP5H_ARATH ATP synthase subunit d, mitochondrial<br>ATPO_ARATH ATP synthase subunit O, mitochondrial<br>RS113_ARATH 40S ribosomal protein S11-3<br>RS163_ARATH 40S ribosomal protein S16-3<br>RS161_ARATH 40S ribosomal protein S16-1<br>RS193_ARATH 40S ribosomal protein S19-3<br>RL322_ARATH 60S ribosomal protein L32-2<br>R10A3_ARATH 60S ribosomal protein L10a-3<br>RLA12_ARATH 60S acidic ribosomal protein P1-2<br>R23A1_ARATH 60S ribosomal protein L23a-1<br>EIF3E_ARATH Eukaryotic translation initiation factor 3 subunit E<br>SYTM1_ARATH Threonine--tRNA ligase, mitochondrial 1<br>SYHC_ARATH Histidine--tRNA ligase, cytoplasmic<br>BTF3L_ARATH Nascent polypeptide-associated complex subunit beta<br>LEU31_ARATH 3-isopropylmalate dehydrogenase 1, chloroplastic<br>LEU32_ARATH 3-isopropylmalate dehydrogenase 2, chloroplastic<br>LEU33_ARATH 3-isopropylmalate dehydrogenase 3, chloroplastic<br>LEUD3_ARATH 3-isopropylmalate dehydratase small subunit 3<br>MAM1_ARATH Methylthioalkylmalate synthase 1, chloroplastic<br>C83B1_ARATH Cytochrome P450 83B1<br>APK1_ARATH Adenylyl-sulfate kinase 1, chloroplastic<br>GSHB_ARATH Glutathione synthetase, chloroplastic<br>NRL1_ARATH Nitrilase 1<br>TRPB1_ARATH Tryptophan synthase beta chain 1, chloroplastic<br>HIS2_ARATH Histidine biosynthesis bifunctional protein hisIE, chloroplastic<br>GLUT1_ARATH Glutamate synthase 1 [NADH], chloroplastic<br>GPP2_ARATH (DL)-glycerol-3-phosphatase 2<br>HIS4_ARATH Imidazole glycerol phosphate synthase hisHF, chloroplastic<br>PMM_ARATH Phosphomannomutase<br>ILVB_ARATH Acetolactate synthase, chloroplastic<br>DHQS_ARATH 3-dehydroquinate synthase, chloroplastic<br>KDSA1_ARATH 2-dehydro-3-deoxyphosphooctonate aldolase 1<br>GMD1_ARATH GDP-mannose 4,6 dehydratase 1<br>KPRS5_ARATH Ribose-phosphate pyrophosphokinase 5, chloroplastic<br>PDX13_ARATH Pyridoxal 5'-phosphate synthase subunit PDX1.3<br>ATPA_ARATH ATP synthase subunit alpha, chloroplastic<br>RMLCD_ARATH Bifunctional dTDP-4-dehydrorhamnose 3,5-epimerase/dTDP-4-dehydrorhamnose reductase

						APT3_ARATH Adenine phosphoribosyltransferase 3<br>SERA2_ARATH D-3-phosphoglycerate dehydrogenase 2, chloroplastic<br>PYRC_ARATH Dihydroorotase, mitochondrial<br>GPP1_ARATH (DL)-glycerol-3-phosphatase 1, mitochondrial<br>AOC4_ARATH Allene oxide cyclase 4, chloroplastic<br>IMDH2_ARATH Inosine-5'-monophosphate dehydrogenase 2<br>H2AV3_ARATH Probable histone H2A variant 3<br>FOLD2_ARATH Bifunctional protein FolD 2<br>MBD10_ARATH Methyl-CpG-binding domain-containing protein 10

GO:0044281	small molecule metabolic process	GO:0044710	single-organism metabolic process	33.1	84	SOX_ARATH Probable sarcosine oxidase<br>GLO2C_ARATH Hydroxyacylglutathione hydrolase cytoplasmic<br>JAL28_ARATH Nitrile-specifier protein 1<br>ITPK1_ARATH Inositol-tetrakisphosphate 1-kinase 1<br>UGE1_ARATH Bifunctional UDP-glucose 4-epimerase and UDP-xylose 4-epimerase 1<br>ATP5H_ARATH ATP synthase subunit d, mitochondrial<br>ATPO_ARATH ATP synthase subunit O, mitochondrial<br>SYTM1_ARATH Threonine--tRNA ligase, mitochondrial 1<br>SYHC_ARATH Histidine--tRNA ligase, cytoplasmic<br>LEU31_ARATH 3-isopropylmalate dehydrogenase 1, chloroplastic<br>LEU32_ARATH 3-isopropylmalate dehydrogenase 2, chloroplastic<br>LEU33_ARATH 3-isopropylmalate dehydrogenase 3, chloroplastic<br>LEUD3_ARATH 3-isopropylmalate dehydratase small subunit 3<br>C83B1_ARATH Cytochrome P450 83B1<br>IGMT1_ARATH Indole glucosinolate O-methyltransferase 1<br>APK1_ARATH Adenylyl-sulfate kinase 1, chloroplastic<br>GSHB_ARATH Glutathione synthetase, chloroplastic<br>NRL1_ARATH Nitrilase 1<br>TRPB1_ARATH Tryptophan synthase beta chain 1, chloroplastic<br>FAAH_ARATH Fatty acid amide hydrolase<br>BGL22_ARATH Beta-glucosidase 22<br>BGL21_ARATH Beta-glucosidase 21<br>HIS2_ARATH Histidine biosynthesis bifunctional protein hisIE, chloroplastic<br>G6PD2_ARATH Glucose-6-phosphate 1-dehydrogenase 2, chloroplastic<br>GLUT1_ARATH Glutamate synthase 1 [NADH], chloroplastic<br>GPP2_ARATH (DL)-glycerol-3-phosphatase 2<br>HIS4_ARATH Imidazole glycerol phosphate synthase hisHF, chloroplastic<br>PMM_ARATH Phosphomannomutase<br>ILVB_ARATH Acetolactate synthase, chloroplastic<br>GCSP2_ARATH Glycine dehydrogenase (decarboxylating) 2, mitochondrial<br>PLDA1_ARATH Phospholipase D alpha 1<br>DHQS_ARATH 3-dehydroquinate synthase, chloroplastic<br>KDSA1_ARATH 2-dehydro-3-deoxyphosphooctonate aldolase 1<br>DCI1_ARATH Delta(3,5)-Delta(2,4)-dienoyl-CoA isomerase, peroxisomal<br>THIC2_ARATH Probable acetyl-CoA acetyltransferase, cytosolic 2<br>ODO2A_ARATH Dihydrolipoyllysine-residue succinyltransferase component of 2-oxoglutarate dehydrogenase complex 1, mitochondrial<br>MAO1_ARATH NAD-dependent malic enzyme 1, mitochondrial<br>KPRS5_ARATH Ribose-phosphate pyrophosphokinase 5, chloroplastic<br>ODO2B_ARATH Dihydrolipoyllysine-residue succinyltransferase component of 2-oxoglutarate dehydrogenase complex 2, mitochondrial<br>PDX13_ARATH Pyridoxal 5'-phosphate synthase subunit PDX1.3<br>ATPA_ARATH ATP synthase subunit alpha, chloroplastic<br>APT3_ARATH Adenine phosphoribosyltransferase 3

						SERA2_ARATH D-3-phosphoglycerate dehydrogenase 2, chloroplastic<br>FUM1_ARATH Fumarate hydratase 1, mitochondrial<br>PYRC_ARATH Dihydroorotase, mitochondrial<br>GPP1_ARATH (DL)-glycerol-3-phosphatase 1, mitochondrial<br>AOC4_ARATH Allene oxide cyclase 4, chloroplastic<br>IMDH2_ARATH Inosine-5'-monophosphate dehydrogenase 2<br>LGUC_ARATH Probable lactoylglutathione lyase, chloroplastic<br>GCSP1_ARATH Glycine dehydrogenase (decarboxylating) 1, mitochondrial<br>FOLD2_ARATH Bifunctional protein FolD 2

GO:0034641	cellular nitrogen compound metabolic process	GO:0006807, GO:0044237	nitrogen compound metabolic process, cellular metabolic process	22.55	76	SOX_ARATH Probable sarcosine oxidase<br>JAL28_ARATH Nitrile-specifier protein 1<br>UGE1_ARATH Bifunctional UDP-glucose 4-epimerase and UDP-xylose 4-epimerase 1<br>ATP5H_ARATH ATP synthase subunit d, mitochondrial<br>ATPO_ARATH ATP synthase subunit O, mitochondrial<br>SYTM1_ARATH Threonine--tRNA ligase, mitochondrial 1<br>SYHC_ARATH Histidine--tRNA ligase, cytoplasmic<br>C83B1_ARATH Cytochrome P450 83B1<br>IGMT1_ARATH Indole glucosinolate O-methyltransferase 1<br>GSHB_ARATH Glutathione synthetase, chloroplastic<br>NRL1_ARATH Nitrilase 1<br>TRPB1_ARATH Tryptophan synthase beta chain 1, chloroplastic<br>FAAH_ARATH Fatty acid amide hydrolase<br>HIS2_ARATH Histidine biosynthesis bifunctional protein hisIE, chloroplastic<br>G6PD2_ARATH Glucose-6-phosphate 1-dehydrogenase 2, chloroplastic<br>HIS4_ARATH Imidazole glycerol phosphate synthase hisHF, chloroplastic<br>PMM_ARATH Phosphomannomutase<br>KPRS5_ARATH Ribose-phosphate pyrophosphokinase 5, chloroplastic<br>PDX13_ARATH Pyridoxal 5'-phosphate synthase subunit PDX1.3<br>ATPA_ARATH ATP synthase subunit alpha, chloroplastic<br>APT3_ARATH Adenine phosphoribosyltransferase 3<br>PYRC_ARATH Dihydroorotase, mitochondrial<br>IMDH2_ARATH Inosine-5'-monophosphate dehydrogenase 2<br>FOLD2_ARATH Bifunctional protein FolD 2<br>GSTF7_ARATH Glutathione S-transferase F7<br>PCNA1_ARATH Proliferating cellular nuclear antigen 1<br>PREP1_ARATH Presequence protease 1, chloroplastic/mitochondrial<br>UEV1C_ARATH Ubiquitin-conjugating enzyme E2 variant 1C<br>RBG3_ARATH Glycine-rich RNA-binding protein 3, mitochondrial<br>RS113_ARATH 40S ribosomal protein S11-3<br>RS163_ARATH 40S ribosomal protein S16-3<br>RS161_ARATH 40S ribosomal protein S16-1<br>RS193_ARATH 40S ribosomal protein S19-3<br>RL322_ARATH 60S ribosomal protein L32-2<br>R10A3_ARATH 60S ribosomal protein L10a-3<br>RLA12_ARATH 60S acidic ribosomal protein P1-2<br>R23A1_ARATH 60S ribosomal protein L23a-1<br>EIF3E_ARATH Eukaryotic translation initiation factor 3 subunit E<br>RH15_ARATH DEAD-box ATP-dependent RNA helicase 15<br>BTF3L_ARATH Nascent polypeptide-associated complex subunit beta<br>GSTF9_ARATH Glutathione S-transferase F9<br>GSTF6_ARATH Glutathione S-transferase F6<br>UGPA1_ARATH UTP--glucose-1-phosphate uridylyltransferase 1<br>GMD1_ARATH GDP-mannose 4,6 dehydratase 1

						RMLCD_ARATH Bifunctional dTDP-4-dehydrorhamnose 3,5-epimerase/dTDP-4-dehydrorhamnose reductase<br>H2AV3_ARATH Probable histone H2A variant 3<br>MBD10_ARATH Methyl-CpG-binding domain-containing protein 10

GO:0055114	oxidation-reduction process	GO:0044710	single-organism metabolic process	66.43	65	SOX_ARATH Probable sarcosine oxidase<br>C83B1_ARATH Cytochrome P450 83B1<br>G6PD2_ARATH Glucose-6-phosphate 1-dehydrogenase 2, chloroplastic<br>IMDH2_ARATH Inosine-5'-monophosphate dehydrogenase 2<br>FOLD2_ARATH Bifunctional protein FolD 2<br>GSTF9_ARATH Glutathione S-transferase F9<br>UGPA1_ARATH UTP--glucose-1-phosphate uridylyltransferase 1<br>RMLCD_ARATH Bifunctional dTDP-4-dehydrorhamnose 3,5-epimerase/dTDP-4-dehydrorhamnose reductase<br>LEU31_ARATH 3-isopropylmalate dehydrogenase 1, chloroplastic<br>LEU32_ARATH 3-isopropylmalate dehydrogenase 2, chloroplastic<br>LEU33_ARATH 3-isopropylmalate dehydrogenase 3, chloroplastic<br>GLUT1_ARATH Glutamate synthase 1 [NADH], chloroplastic<br>GCSP2_ARATH Glycine dehydrogenase (decarboxylating) 2, mitochondrial<br>DCI1_ARATH Delta(3,5)-Delta(2,4)-dienoyl-CoA isomerase, peroxisomal<br>THIC2_ARATH Probable acetyl-CoA acetyltransferase, cytosolic 2<br>ODO2A_ARATH Dihydrolipoyllysine-residue succinyltransferase component of 2-oxoglutarate dehydrogenase complex 1, mitochondrial<br>MAO1_ARATH NAD-dependent malic enzyme 1, mitochondrial<br>ODO2B_ARATH Dihydrolipoyllysine-residue succinyltransferase component of 2-oxoglutarate dehydrogenase complex 2, mitochondrial<br>SERA2_ARATH D-3-phosphoglycerate dehydrogenase 2, chloroplastic<br>FUM1_ARATH Fumarate hydratase 1, mitochondrial<br>GCSP1_ARATH Glycine dehydrogenase (decarboxylating) 1, mitochondrial<br>APXS_ARATH L-ascorbate peroxidase S, chloroplastic/mitochondrial<br>MSRB6_ARATH Peptide methionine sulfoxide reductase B6<br>PER57_ARATH Peroxidase 57<br>PER30_ARATH Peroxidase 30<br>PER22_ARATH Peroxidase 22<br>PER15_ARATH Peroxidase 15<br>PER42_ARATH Peroxidase 42<br>NDADB_ARATH NADH dehydrogenase [ubiquinone] 1 alpha subcomplex subunit 13-B<br>CX6B1_ARATH Cytochrome c oxidase subunit 6b-1<br>ALKR2_ARATH Probable aldo-keto reductase 2<br>DHSO_ARATH Sorbitol dehydrogenase<br>AL2B7_ARATH Aldehyde dehydrogenase family 2 member B7, mitochondrial<br>BADH2_ARATH Betaine aldehyde dehydrogenase 2, mitochondrial

GO:0006082	organic acid metabolic process	GO:0044281, GO:0071704, GO:0044237, GO:0044763	small molecule metabolic process, organic substance metabolic process, cellular metabolic process, single-organism cellular process	36.73	60	SOX_ARATH Probable sarcosine oxidase<br>JAL28_ARATH Nitrile-specifier protein 1<br>SYTM1_ARATH Threonine--tRNA ligase, mitochondrial 1<br>SYHC_ARATH Histidine--tRNA ligase, cytoplasmic<br>C83B1_ARATH Cytochrome P450 83B1<br>IGMT1_ARATH Indole glucosinolate O-methyltransferase 1<br>GSHB_ARATH Glutathione synthetase, chloroplastic<br>NRL1_ARATH Nitrilase 1<br>TRPB1_ARATH Tryptophan synthase beta chain 1, chloroplastic<br>HIS2_ARATH Histidine biosynthesis bifunctional protein hisIE, chloroplastic<br>HIS4_ARATH Imidazole glycerol phosphate synthase hisHF, chloroplastic<br>PMM_ARATH Phosphomannomutase<br>PDX13_ARATH Pyridoxal 5'-phosphate synthase subunit PDX1.3<br>FOLD2_ARATH Bifunctional protein FolD 2<br>GLO2C_ARATH Hydroxyacylglutathione hydrolase cytoplasmic<br>LEU31_ARATH 3-isopropylmalate dehydrogenase 1, chloroplastic<br>LEU32_ARATH 3-isopropylmalate dehydrogenase 2, chloroplastic<br>LEU33_ARATH 3-isopropylmalate dehydrogenase 3, chloroplastic<br>LEUD3_ARATH 3-isopropylmalate dehydratase small subunit 3<br>APK1_ARATH Adenylyl-sulfate kinase 1, chloroplastic<br>BGL22_ARATH Beta-glucosidase 22<br>BGL21_ARATH Beta-glucosidase 21<br>GLUT1_ARATH Glutamate synthase 1 [NADH], chloroplastic<br>ILVB_ARATH Acetolactate synthase, chloroplastic<br>GCSP2_ARATH Glycine dehydrogenase (decarboxylating) 2, mitochondrial<br>PLDA1_ARATH Phospholipase D alpha 1<br>DHQS_ARATH 3-dehydroquinate synthase, chloroplastic<br>KDSA1_ARATH 2-dehydro-3-deoxyphosphooctonate aldolase 1<br>DCI1_ARATH Delta(3,5)-Delta(2,4)-dienoyl-CoA isomerase, peroxisomal<br>THIC2_ARATH Probable acetyl-CoA acetyltransferase, cytosolic 2<br>ODO2A_ARATH Dihydrolipoyllysine-residue succinyltransferase component of 2-oxoglutarate dehydrogenase complex 1, mitochondrial<br>MAO1_ARATH NAD-dependent malic enzyme 1, mitochondrial<br>ODO2B_ARATH Dihydrolipoyllysine-residue succinyltransferase component of 2-oxoglutarate dehydrogenase complex 2, mitochondrial<br>SERA2_ARATH D-3-phosphoglycerate dehydrogenase 2, chloroplastic<br>FUM1_ARATH Fumarate hydratase 1, mitochondrial<br>AOC4_ARATH Allene oxide cyclase 4, chloroplastic<br>LGUC_ARATH Probable lactoylglutathione lyase, chloroplastic<br>GCSP1_ARATH Glycine dehydrogenase (decarboxylating) 1, mitochondrial<br>MAM1_ARATH Methylthioalkylmalate synthase 1, chloroplastic

GO:0043170	macromolecule metabolic process	GO:0071704	organic substance metabolic process	28.04	59	UGE1_ARATH Bifunctional UDP-glucose 4-epimerase and UDP-xylose 4-epimerase 1<br>UGPA1_ARATH UTP--glucose-1-phosphate uridylyltransferase 1<br>MSRB6_ARATH Peptide methionine sulfoxide reductase B6<br>PCNA1_ARATH Proliferating cellular nuclear antigen 1<br>VPS29_ARATH Vacuolar protein sorting-associated protein 29<br>CLPP4_ARATH ATP-dependent Clp protease proteolytic subunit 4, chloroplastic<br>CLPP2_ARATH ATP-dependent Clp protease proteolytic subunit 2, mitochondrial<br>PREP1_ARATH Presequence protease 1, chloroplastic/mitochondrial<br>SBT17_ARATH Subtilisin-like protease SBT1.7<br>MAP22_ARATH Methionine aminopeptidase 2B<br>XCP2_ARATH Cysteine protease XCP2<br>TPPII_ARATH Tripeptidyl-peptidase 2<br>UEV1C_ARATH Ubiquitin-conjugating enzyme E2 variant 1C<br>PRS6B_ARATH 26S proteasome regulatory subunit 6B homolog<br>PS12A_ARATH 26S proteasome non-ATPase regulatory subunit 12 homolog A<br>CP20A_ARATH Peptidyl-prolyl cis-trans isomerase CYP20-1<br>RBG3_ARATH Glycine-rich RNA-binding protein 3, mitochondrial<br>RS113_ARATH 40S ribosomal protein S11-3<br>RS163_ARATH 40S ribosomal protein S16-3<br>RS161_ARATH 40S ribosomal protein S16-1<br>RS193_ARATH 40S ribosomal protein S19-3<br>RL322_ARATH 60S ribosomal protein L32-2<br>R10A3_ARATH 60S ribosomal protein L10a-3<br>RLA12_ARATH 60S acidic ribosomal protein P1-2<br>R23A1_ARATH 60S ribosomal protein L23a-1<br>EIF3E_ARATH Eukaryotic translation initiation factor 3 subunit E<br>SYTM1_ARATH Threonine--tRNA ligase, mitochondrial 1<br>SYHC_ARATH Histidine--tRNA ligase, cytoplasmic<br>RH15_ARATH DEAD-box ATP-dependent RNA helicase 15<br>BTF3L_ARATH Nascent polypeptide-associated complex subunit beta<br>PMM_ARATH Phosphomannomutase<br>KDSA1_ARATH 2-dehydro-3-deoxyphosphooctonate aldolase 1<br>H2AV3_ARATH Probable histone H2A variant 3<br>FOLD2_ARATH Bifunctional protein FolD 2<br>MBD10_ARATH Methyl-CpG-binding domain-containing protein 10

GO:1901360	organic cyclic compound metabolic process	GO:0071704	organic substance metabolic process	20.14	54	UGE1_ARATH Bifunctional UDP-glucose 4-epimerase and UDP-xylose 4-epimerase 1<br>SYTM1_ARATH Threonine--tRNA ligase, mitochondrial 1<br>SYHC_ARATH Histidine--tRNA ligase, cytoplasmic<br>PMM_ARATH Phosphomannomutase<br>FOLD2_ARATH Bifunctional protein FolD 2<br>PCNA1_ARATH Proliferating cellular nuclear antigen 1<br>PREP1_ARATH Presequence protease 1, chloroplastic/mitochondrial<br>UEV1C_ARATH Ubiquitin-conjugating enzyme E2 variant 1C<br>RBG3_ARATH Glycine-rich RNA-binding protein 3, mitochondrial<br>RS113_ARATH 40S ribosomal protein S11-3<br>RS163_ARATH 40S ribosomal protein S16-3<br>RS161_ARATH 40S ribosomal protein S16-1<br>RS193_ARATH 40S ribosomal protein S19-3<br>RL322_ARATH 60S ribosomal protein L32-2<br>R10A3_ARATH 60S ribosomal protein L10a-3<br>RLA12_ARATH 60S acidic ribosomal protein P1-2<br>R23A1_ARATH 60S ribosomal protein L23a-1<br>EIF3E_ARATH Eukaryotic translation initiation factor 3 subunit E<br>RH15_ARATH DEAD-box ATP-dependent RNA helicase 15<br>BTF3L_ARATH Nascent polypeptide-associated complex subunit beta<br>UGPA1_ARATH UTP--glucose-1-phosphate uridylyltransferase 1<br>H2AV3_ARATH Probable histone H2A variant 3<br>MBD10_ARATH Methyl-CpG-binding domain-containing protein 10<br>KDSA1_ARATH 2-dehydro-3-deoxyphosphooctonate aldolase 1<br>MSRB6_ARATH Peptide methionine sulfoxide reductase B6<br>VPS29_ARATH Vacuolar protein sorting-associated protein 29<br>CLPP4_ARATH ATP-dependent Clp protease proteolytic subunit 4, chloroplastic<br>CLPP2_ARATH ATP-dependent Clp protease proteolytic subunit 2, mitochondrial<br>SBT17_ARATH Subtilisin-like protease SBT1.7<br>MAP22_ARATH Methionine aminopeptidase 2B<br>XCP2_ARATH Cysteine protease XCP2<br>TPPII_ARATH Tripeptidyl-peptidase 2<br>PRS6B_ARATH 26S proteasome regulatory subunit 6B homolog<br>PS12A_ARATH 26S proteasome non-ATPase regulatory subunit 12 homolog A<br>CP20A_ARATH Peptidyl-prolyl cis-trans isomerase CYP20-1

GO:0006725	cellular aromatic compound metabolic process	GO:0044237	cellular metabolic process	42.58	54	SOX_ARATH Probable sarcosine oxidase<br>UGE1_ARATH Bifunctional UDP-glucose 4-epimerase and UDP-xylose 4-epimerase 1<br>ATP5H_ARATH ATP synthase subunit d, mitochondrial<br>ATPO_ARATH ATP synthase subunit O, mitochondrial<br>SYTM1_ARATH Threonine--tRNA ligase, mitochondrial 1<br>SYHC_ARATH Histidine--tRNA ligase, cytoplasmic<br>C83B1_ARATH Cytochrome P450 83B1<br>IGMT1_ARATH Indole glucosinolate O-methyltransferase 1<br>NRL1_ARATH Nitrilase 1<br>TRPB1_ARATH Tryptophan synthase beta chain 1, chloroplastic<br>HIS2_ARATH Histidine biosynthesis bifunctional protein hisIE, chloroplastic<br>G6PD2_ARATH Glucose-6-phosphate 1-dehydrogenase 2, chloroplastic<br>HIS4_ARATH Imidazole glycerol phosphate synthase hisHF, chloroplastic<br>PMM_ARATH Phosphomannomutase<br>KPRS5_ARATH Ribose-phosphate pyrophosphokinase 5, chloroplastic<br>PDX13_ARATH Pyridoxal 5'-phosphate synthase subunit PDX1.3<br>ATPA_ARATH ATP synthase subunit alpha, chloroplastic<br>APT3_ARATH Adenine phosphoribosyltransferase 3<br>PYRC_ARATH Dihydroorotase, mitochondrial<br>IMDH2_ARATH Inosine-5'-monophosphate dehydrogenase 2<br>FOLD2_ARATH Bifunctional protein FolD 2<br>PCNA1_ARATH Proliferating cellular nuclear antigen 1<br>PREP1_ARATH Presequence protease 1, chloroplastic/mitochondrial<br>UEV1C_ARATH Ubiquitin-conjugating enzyme E2 variant 1C<br>RBG3_ARATH Glycine-rich RNA-binding protein 3, mitochondrial<br>EIF3E_ARATH Eukaryotic translation initiation factor 3 subunit E<br>RH15_ARATH DEAD-box ATP-dependent RNA helicase 15<br>BTF3L_ARATH Nascent polypeptide-associated complex subunit beta<br>GMD1_ARATH GDP-mannose 4,6 dehydratase 1<br>RMLCD_ARATH Bifunctional dTDP-4-dehydrorhamnose 3,5-epimerase/dTDP-4-dehydrorhamnose reductase<br>H2AV3_ARATH Probable histone H2A variant 3<br>MBD10_ARATH Methyl-CpG-binding domain-containing protein 10<br>BGL21_ARATH Beta-glucosidase 21<br>DIR13_ARATH Dirigent protein 13

GO:0046483	heterocycle metabolic process	GO:0044237	cellular metabolic process	18.46	52	SOX_ARATH Probable sarcosine oxidase<br>UGE1_ARATH Bifunctional UDP-glucose 4-epimerase and UDP-xylose 4-epimerase 1<br>ATP5H_ARATH ATP synthase subunit d, mitochondrial<br>ATPO_ARATH ATP synthase subunit O, mitochondrial<br>SYTM1_ARATH Threonine--tRNA ligase, mitochondrial 1<br>SYHC_ARATH Histidine--tRNA ligase, cytoplasmic<br>C83B1_ARATH Cytochrome P450 83B1<br>IGMT1_ARATH Indole glucosinolate O-methyltransferase 1<br>NRL1_ARATH Nitrilase 1<br>TRPB1_ARATH Tryptophan synthase beta chain 1, chloroplastic<br>HIS2_ARATH Histidine biosynthesis bifunctional protein hisIE, chloroplastic<br>G6PD2_ARATH Glucose-6-phosphate 1-dehydrogenase 2, chloroplastic<br>HIS4_ARATH Imidazole glycerol phosphate synthase hisHF, chloroplastic<br>PMM_ARATH Phosphomannomutase<br>KPRS5_ARATH Ribose-phosphate pyrophosphokinase 5, chloroplastic<br>PDX13_ARATH Pyridoxal 5'-phosphate synthase subunit PDX1.3<br>ATPA_ARATH ATP synthase subunit alpha, chloroplastic<br>APT3_ARATH Adenine phosphoribosyltransferase 3<br>PYRC_ARATH Dihydroorotase, mitochondrial<br>IMDH2_ARATH Inosine-5'-monophosphate dehydrogenase 2<br>FOLD2_ARATH Bifunctional protein FolD 2<br>PCNA1_ARATH Proliferating cellular nuclear antigen 1<br>PREP1_ARATH Presequence protease 1, chloroplastic/mitochondrial<br>UEV1C_ARATH Ubiquitin-conjugating enzyme E2 variant 1C<br>RBG3_ARATH Glycine-rich RNA-binding protein 3, mitochondrial<br>EIF3E_ARATH Eukaryotic translation initiation factor 3 subunit E<br>RH15_ARATH DEAD-box ATP-dependent RNA helicase 15<br>BTF3L_ARATH Nascent polypeptide-associated complex subunit beta<br>UGPA1_ARATH UTP--glucose-1-phosphate uridylyltransferase 1<br>GMD1_ARATH GDP-mannose 4,6 dehydratase 1<br>RMLCD_ARATH Bifunctional dTDP-4-dehydrorhamnose 3,5-epimerase/dTDP-4-dehydrorhamnose reductase<br>H2AV3_ARATH Probable histone H2A variant 3<br>MBD10_ARATH Methyl-CpG-binding domain-containing protein 10<br>BGL21_ARATH Beta-glucosidase 21

GO:0044711	single-organism biosynthetic process	GO:0009058, GO:0044710	biosynthetic process, single-organism metabolic process	16.63	51	UGE1_ARATH Bifunctional UDP-glucose 4-epimerase and UDP-xylose 4-epimerase 1<br>ATP5H_ARATH ATP synthase subunit d, mitochondrial<br>ATPO_ARATH ATP synthase subunit O, mitochondrial<br>C83B1_ARATH Cytochrome P450 83B1<br>NRL1_ARATH Nitrilase 1<br>TRPB1_ARATH Tryptophan synthase beta chain 1, chloroplastic<br>HIS2_ARATH Histidine biosynthesis bifunctional protein hisIE, chloroplastic<br>HIS4_ARATH Imidazole glycerol phosphate synthase hisHF, chloroplastic<br>PMM_ARATH Phosphomannomutase<br>KPRS5_ARATH Ribose-phosphate pyrophosphokinase 5, chloroplastic<br>PDX13_ARATH Pyridoxal 5'-phosphate synthase subunit PDX1.3<br>ATPA_ARATH ATP synthase subunit alpha, chloroplastic<br>APT3_ARATH Adenine phosphoribosyltransferase 3<br>PYRC_ARATH Dihydroorotase, mitochondrial<br>IMDH2_ARATH Inosine-5'-monophosphate dehydrogenase 2<br>FOLD2_ARATH Bifunctional protein FolD 2<br>ITPK1_ARATH Inositol-tetrakisphosphate 1-kinase 1<br>LEU31_ARATH 3-isopropylmalate dehydrogenase 1, chloroplastic<br>LEU32_ARATH 3-isopropylmalate dehydrogenase 2, chloroplastic<br>LEU33_ARATH 3-isopropylmalate dehydrogenase 3, chloroplastic<br>LEUD3_ARATH 3-isopropylmalate dehydratase small subunit 3<br>APK1_ARATH Adenylyl-sulfate kinase 1, chloroplastic<br>GLUT1_ARATH Glutamate synthase 1 [NADH], chloroplastic<br>GPP2_ARATH (DL)-glycerol-3-phosphatase 2<br>ILVB_ARATH Acetolactate synthase, chloroplastic<br>DHQS_ARATH 3-dehydroquinate synthase, chloroplastic<br>KDSA1_ARATH 2-dehydro-3-deoxyphosphooctonate aldolase 1<br>SERA2_ARATH D-3-phosphoglycerate dehydrogenase 2, chloroplastic<br>GPP1_ARATH (DL)-glycerol-3-phosphatase 1, mitochondrial<br>AOC4_ARATH Allene oxide cyclase 4, chloroplastic<br>DIR13_ARATH Dirigent protein 13<br>MAM1_ARATH Methylthioalkylmalate synthase 1, chloroplastic7

GO:0019538	protein metabolic process	GO:0044238, GO:0043170	primary metabolic process, macromolecule metabolic process	27.64	47	SYTM1_ARATH Threonine--tRNA ligase, mitochondrial 1<br>SYHC_ARATH Histidine--tRNA ligase, cytoplasmic<br>PMM_ARATH Phosphomannomutase<br>PREP1_ARATH Presequence protease 1, chloroplastic/mitochondrial<br>UEV1C_ARATH Ubiquitin-conjugating enzyme E2 variant 1C<br>RS113_ARATH 40S ribosomal protein S11-3<br>RS163_ARATH 40S ribosomal protein S16-3<br>RS161_ARATH 40S ribosomal protein S16-1<br>RS193_ARATH 40S ribosomal protein S19-3<br>RL322_ARATH 60S ribosomal protein L32-2<br>R10A3_ARATH 60S ribosomal protein L10a-3<br>RLA12_ARATH 60S acidic ribosomal protein P1-2<br>R23A1_ARATH 60S ribosomal protein L23a-1<br>EIF3E_ARATH Eukaryotic translation initiation factor 3 subunit E<br>MSRB6_ARATH Peptide methionine sulfoxide reductase B6<br>CLPP4_ARATH ATP-dependent Clp protease proteolytic subunit 4, chloroplastic<br>CLPP2_ARATH ATP-dependent Clp protease proteolytic subunit 2, mitochondrial<br>SBT17_ARATH Subtilisin-like protease SBT1.7<br>MAP22_ARATH Methionine aminopeptidase 2B<br>XCP2_ARATH Cysteine protease XCP2<br>TPPII_ARATH Tripeptidyl-peptidase 2<br>PRS6B_ARATH 26S proteasome regulatory subunit 6B homolog<br>PS12A_ARATH 26S proteasome non-ATPase regulatory subunit 12 homolog A<br>CP20A_ARATH Peptidyl-prolyl cis-trans isomerase CYP20-1

GO:0044260	cellular macromolecule metabolic process	GO:0043170, GO:0044237	macromolecule metabolic process, cellular metabolic process	18.37	45	UGE1_ARATH Bifunctional UDP-glucose 4-epimerase and UDP-xylose 4-epimerase 1<br>SYTM1_ARATH Threonine--tRNA ligase, mitochondrial 1<br>SYHC_ARATH Histidine--tRNA ligase, cytoplasmic<br>PMM_ARATH Phosphomannomutase<br>FOLD2_ARATH Bifunctional protein FolD 2<br>PCNA1_ARATH Proliferating cellular nuclear antigen 1<br>PREP1_ARATH Presequence protease 1, chloroplastic/mitochondrial<br>UEV1C_ARATH Ubiquitin-conjugating enzyme E2 variant 1C<br>RBG3_ARATH Glycine-rich RNA-binding protein 3, mitochondrial<br>RS113_ARATH 40S ribosomal protein S11-3<br>RS163_ARATH 40S ribosomal protein S16-3<br>RS161_ARATH 40S ribosomal protein S16-1<br>RS193_ARATH 40S ribosomal protein S19-3<br>RL322_ARATH 60S ribosomal protein L32-2<br>R10A3_ARATH 60S ribosomal protein L10a-3<br>RLA12_ARATH 60S acidic ribosomal protein P1-2<br>R23A1_ARATH 60S ribosomal protein L23a-1<br>EIF3E_ARATH Eukaryotic translation initiation factor 3 subunit E<br>RH15_ARATH DEAD-box ATP-dependent RNA helicase 15<br>BTF3L_ARATH Nascent polypeptide-associated complex subunit beta<br>UGPA1_ARATH UTP--glucose-1-phosphate uridylyltransferase 1<br>H2AV3_ARATH Probable histone H2A variant 3<br>MBD10_ARATH Methyl-CpG-binding domain-containing protein 10<br>KDSA1_ARATH 2-dehydro-3-deoxyphosphooctonate aldolase 1<br>MSRB6_ARATH Peptide methionine sulfoxide reductase B6<br>VPS29_ARATH Vacuolar protein sorting-associated protein 29<br>XCP2_ARATH Cysteine protease XCP2<br>PRS6B_ARATH 26S proteasome regulatory subunit 6B homolog<br>PS12A_ARATH 26S proteasome non-ATPase regulatory subunit 12 homolog A<br>CP20A_ARATH Peptidyl-prolyl cis-trans isomerase CYP20-1

GO:0006810	transport	GO:0051234	establishment of localization	91.48	42	ATP5H_ARATH ATP synthase subunit d, mitochondrial<br>ATPO_ARATH ATP synthase subunit O, mitochondrial<br>PMM_ARATH Phosphomannomutase<br>ATPA_ARATH ATP synthase subunit alpha, chloroplastic<br>RH15_ARATH DEAD-box ATP-dependent RNA helicase 15<br>PIP12_ARATH Aquaporin PIP1-2<br>PIP21_ARATH Aquaporin PIP2-1<br>VATD_ARATH V-type proton ATPase subunit D<br>VA0D2_ARATH V-type proton ATPase subunit d2<br>SC13A_ARATH Protein transport protein SEC13 homolog A<br>VAP12_ARATH Vesicle-associated protein 1-2<br>SNAA2_ARATH Alpha-soluble NSF attachment protein 2<br>VPS29_ARATH Vacuolar protein sorting-associated protein 29<br>HLB1_ARATH Protein HLB1<br>SYP32_ARATH Syntaxin-32<br>SYT1_ARATH Synaptotagmin-1<br>PATL4_ARATH Patellin-4<br>TI232_ARATH Mitochondrial import inner membrane translocase subunit TIM23-2<br>TIM13_ARATH Mitochondrial import inner membrane translocase subunit TIM13<br>PUMP1_ARATH Mitochondrial uncoupling protein 1<br>ADT2_ARATH ADP,ATP carrier protein 2, mitochondrial<br>HS905_ARATH Heat shock protein 90-5, chloroplastic<br>AB1F_ARATH ABC transporter F family member 1<br>NACA1_ARATH Nascent polypeptide-associated complex subunit alpha-like protein 1<br>NACA3_ARATH Nascent polypeptide-associated complex subunit alpha-like protein 3<br>IMPA6_ARATH Importin subunit alpha-6<br>RAGP1_ARATH RAN GTPase-activating protein 1<br>BIG_ARATH Auxin transport protein BIG

GO:0006139	nucleobase-containing compound metabolic process	GO:0044238, GO:0034641, GO:0046483, GO:0006725, GO:1901360	primary metabolic process, cellular nitrogen compound metabolic process, heterocycle metabolic process, cellular aromatic compound metabolic process, organic cyclic compound metabolic process	17.25	38	UGE1_ARATH Bifunctional UDP-glucose 4-epimerase and UDP-xylose 4-epimerase 1<br>ATP5H_ARATH ATP synthase subunit d, mitochondrial<br>ATPO_ARATH ATP synthase subunit O, mitochondrial<br>SYTM1_ARATH Threonine--tRNA ligase, mitochondrial 1<br>SYHC_ARATH Histidine--tRNA ligase, cytoplasmic<br>G6PD2_ARATH Glucose-6-phosphate 1-dehydrogenase 2, chloroplastic<br>PMM_ARATH Phosphomannomutase<br>KPRS5_ARATH Ribose-phosphate pyrophosphokinase 5, chloroplastic<br>ATPA_ARATH ATP synthase subunit alpha, chloroplastic<br>APT3_ARATH Adenine phosphoribosyltransferase 3<br>PYRC_ARATH Dihydroorotase, mitochondrial<br>IMDH2_ARATH Inosine-5'-monophosphate dehydrogenase 2<br>FOLD2_ARATH Bifunctional protein FolD 2<br>PCNA1_ARATH Proliferating cellular nuclear antigen 1<br>PREP1_ARATH Presequence protease 1, chloroplastic/mitochondrial<br>UEV1C_ARATH Ubiquitin-conjugating enzyme E2 variant 1C<br>RBG3_ARATH Glycine-rich RNA-binding protein 3, mitochondrial<br>EIF3E_ARATH Eukaryotic translation initiation factor 3 subunit E<br>RH15_ARATH DEAD-box ATP-dependent RNA helicase 15<br>BTF3L_ARATH Nascent polypeptide-associated complex subunit beta<br>UGPA1_ARATH UTP--glucose-1-phosphate uridylyltransferase 1<br>GMD1_ARATH GDP-mannose 4,6 dehydratase 1<br>RMLCD_ARATH Bifunctional dTDP-4-dehydrorhamnose 3,5-epimerase/dTDP-4-dehydrorhamnose reductase<br>H2AV3_ARATH Probable histone H2A variant 3<br>MBD10_ARATH Methyl-CpG-binding domain-containing protein 10

GO:0006520	cellular amino acid metabolic process	GO:0044238, GO:1901564, GO:0019752	primary metabolic process, organonitrogen compound metabolic process, carboxylic acid metabolic process	45.84	35	SYTM1_ARATH Threonine--tRNA ligase, mitochondrial 1<br>SYHC_ARATH Histidine--tRNA ligase, cytoplasmic<br>LEU31_ARATH 3-isopropylmalate dehydrogenase 1, chloroplastic<br>LEU32_ARATH 3-isopropylmalate dehydrogenase 2, chloroplastic<br>LEU33_ARATH 3-isopropylmalate dehydrogenase 3, chloroplastic<br>LEUD3_ARATH 3-isopropylmalate dehydratase small subunit 3<br>C83B1_ARATH Cytochrome P450 83B1<br>APK1_ARATH Adenylyl-sulfate kinase 1, chloroplastic<br>GSHB_ARATH Glutathione synthetase, chloroplastic<br>TRPB1_ARATH Tryptophan synthase beta chain 1, chloroplastic<br>HIS2_ARATH Histidine biosynthesis bifunctional protein hisIE, chloroplastic<br>GLUT1_ARATH Glutamate synthase 1 [NADH], chloroplastic<br>HIS4_ARATH Imidazole glycerol phosphate synthase hisHF, chloroplastic<br>ILVB_ARATH Acetolactate synthase, chloroplastic<br>GCSP2_ARATH Glycine dehydrogenase (decarboxylating) 2, mitochondrial<br>ODO2A_ARATH Dihydrolipoyllysine-residue succinyltransferase component of 2-oxoglutarate dehydrogenase complex 1, mitochondrial<br>ODO2B_ARATH Dihydrolipoyllysine-residue succinyltransferase component of 2-oxoglutarate dehydrogenase complex 2, mitochondrial<br>PDX13_ARATH Pyridoxal 5'-phosphate synthase subunit PDX1.3<br>SERA2_ARATH D-3-phosphoglycerate dehydrogenase 2, chloroplastic<br>GCSP1_ARATH Glycine dehydrogenase (decarboxylating) 1, mitochondrial

GO:0044248	cellular catabolic process	GO:0009056, GO:0044237	catabolic process, cellular metabolic process	11.55	34	GLO2C_ARATH Hydroxyacylglutathione hydrolase cytoplasmic<br>JAL28_ARATH Nitrile-specifier protein 1<br>APXS_ARATH L-ascorbate peroxidase S, chloroplastic/mitochondrial<br>PER57_ARATH Peroxidase 57<br>PER30_ARATH Peroxidase 30<br>PER22_ARATH Peroxidase 22<br>PER15_ARATH Peroxidase 15<br>PER42_ARATH Peroxidase 42<br>GSTF7_ARATH Glutathione S-transferase F7<br>XCP2_ARATH Cysteine protease XCP2<br>PRS6B_ARATH 26S proteasome regulatory subunit 6B homolog<br>PS12A_ARATH 26S proteasome non-ATPase regulatory subunit 12 homolog A<br>GSTF9_ARATH Glutathione S-transferase F9<br>GSTF6_ARATH Glutathione S-transferase F6<br>BGL22_ARATH Beta-glucosidase 22<br>BGL21_ARATH Beta-glucosidase 21<br>GCSP2_ARATH Glycine dehydrogenase (decarboxylating) 2, mitochondrial<br>DCI1_ARATH Delta(3,5)-Delta(2,4)-dienoyl-CoA isomerase, peroxisomal<br>THIC2_ARATH Probable acetyl-CoA acetyltransferase, cytosolic 2<br>ODO2A_ARATH Dihydrolipoyllysine-residue succinyltransferase component of 2-oxoglutarate dehydrogenase complex 1, mitochondrial<br>ODO2B_ARATH Dihydrolipoyllysine-residue succinyltransferase component of 2-oxoglutarate dehydrogenase complex 2, mitochondrial<br>CINV2_ARATH Alkaline/neutral invertase CINV2<br>LGUC_ARATH Probable lactoylglutathione lyase, chloroplastic<br>GCSP1_ARATH Glycine dehydrogenase (decarboxylating) 1, mitochondrial

GO:1901135	carbohydrate derivative metabolic process	GO:0071704	organic substance metabolic process	9.87	32	JAL28_ARATH Nitrile-specifier protein 1<br>UGE1_ARATH Bifunctional UDP-glucose 4-epimerase and UDP-xylose 4-epimerase 1<br>ATP5H_ARATH ATP synthase subunit d, mitochondrial<br>ATPO_ARATH ATP synthase subunit O, mitochondrial<br>LEU33_ARATH 3-isopropylmalate dehydrogenase 3, chloroplastic<br>LEUD3_ARATH 3-isopropylmalate dehydratase small subunit 3<br>MAM1_ARATH Methylthioalkylmalate synthase 1, chloroplastic<br>C83B1_ARATH Cytochrome P450 83B1<br>IGMT1_ARATH Indole glucosinolate O-methyltransferase 1<br>APK1_ARATH Adenylyl-sulfate kinase 1, chloroplastic<br>BGL22_ARATH Beta-glucosidase 22<br>BGL21_ARATH Beta-glucosidase 21<br>UGPA1_ARATH UTP--glucose-1-phosphate uridylyltransferase 1<br>G6PD2_ARATH Glucose-6-phosphate 1-dehydrogenase 2, chloroplastic<br>PMM_ARATH Phosphomannomutase<br>KDSA1_ARATH 2-dehydro-3-deoxyphosphooctonate aldolase 1<br>GMD1_ARATH GDP-mannose 4,6 dehydratase 1<br>KPRS5_ARATH Ribose-phosphate pyrophosphokinase 5, chloroplastic<br>ATPA_ARATH ATP synthase subunit alpha, chloroplastic<br>RMLCD_ARATH Bifunctional dTDP-4-dehydrorhamnose 3,5-epimerase/dTDP-4-dehydrorhamnose reductase<br>APT3_ARATH Adenine phosphoribosyltransferase 3<br>PYRC_ARATH Dihydroorotase, mitochondrial<br>IMDH2_ARATH Inosine-5'-monophosphate dehydrogenase 2

GO:0006793	phosphorus metabolic process	GO:0044237	cellular metabolic process	23.54	31	ITPK1_ARATH Inositol-tetrakisphosphate 1-kinase 1<br>UGE1_ARATH Bifunctional UDP-glucose 4-epimerase and UDP-xylose 4-epimerase 1<br>ATP5H_ARATH ATP synthase subunit d, mitochondrial<br>ATPO_ARATH ATP synthase subunit O, mitochondrial<br>APK1_ARATH Adenylyl-sulfate kinase 1, chloroplastic<br>UGPA1_ARATH UTP--glucose-1-phosphate uridylyltransferase 1<br>G6PD2_ARATH Glucose-6-phosphate 1-dehydrogenase 2, chloroplastic<br>GPP2_ARATH (DL)-glycerol-3-phosphatase 2<br>PLDA1_ARATH Phospholipase D alpha 1<br>ODO2A_ARATH Dihydrolipoyllysine-residue succinyltransferase component of 2-oxoglutarate dehydrogenase complex 1, mitochondrial<br>GMD1_ARATH GDP-mannose 4,6 dehydratase 1<br>IPYR2_ARATH Soluble inorganic pyrophosphatase 2<br>KPRS5_ARATH Ribose-phosphate pyrophosphokinase 5, chloroplastic<br>ODO2B_ARATH Dihydrolipoyllysine-residue succinyltransferase component of 2-oxoglutarate dehydrogenase complex 2, mitochondrial<br>PDX13_ARATH Pyridoxal 5'-phosphate synthase subunit PDX1.3<br>ATPA_ARATH ATP synthase subunit alpha, chloroplastic<br>RMLCD_ARATH Bifunctional dTDP-4-dehydrorhamnose 3,5-epimerase/dTDP-4-dehydrorhamnose reductase<br>APT3_ARATH Adenine phosphoribosyltransferase 3<br>PYRC_ARATH Dihydroorotase, mitochondrial<br>GPP1_ARATH (DL)-glycerol-3-phosphatase 1, mitochondrial<br>IMDH2_ARATH Inosine-5'-monophosphate dehydrogenase 2

GO:0006970	response to osmotic stress	GO:0006950, GO:0009628	response to stress, response to abiotic stimulus	16.08	24	ATP5H_ARATH ATP synthase subunit d, mitochondrial<br>UGPA1_ARATH UTP--glucose-1-phosphate uridylyltransferase 1<br>PDX13_ARATH Pyridoxal 5'-phosphate synthase subunit PDX1.3<br>PIP12_ARATH Aquaporin PIP1-2<br>OSL3_ARATH Osmotin-like protein OSM34<br>PER22_ARATH Peroxidase 22<br>GSTF7_ARATH Glutathione S-transferase F7<br>NAI2_ARATH TSA1-like protein<br>CX6B1_ARATH Cytochrome c oxidase subunit 6b-1<br>HS905_ARATH Heat shock protein 90-5, chloroplastic<br>EIF3E_ARATH Eukaryotic translation initiation factor 3 subunit E<br>BTF3L_ARATH Nascent polypeptide-associated complex subunit beta<br>NACA1_ARATH Nascent polypeptide-associated complex subunit alpha-like protein 1<br>NACA3_ARATH Nascent polypeptide-associated complex subunit alpha-like protein 3<br>LEU31_ARATH 3-isopropylmalate dehydrogenase 1, chloroplastic<br>LEU33_ARATH 3-isopropylmalate dehydrogenase 3, chloroplastic<br>LEUD3_ARATH 3-isopropylmalate dehydratase small subunit 3<br>GSTF6_ARATH Glutathione S-transferase F6<br>TRPB1_ARATH Tryptophan synthase beta chain 1, chloroplastic<br>BGL22_ARATH Beta-glucosidase 22<br>BGL21_ARATH Beta-glucosidase 21<br>PMM_ARATH Phosphomannomutase<br>MAO1_ARATH NAD-dependent malic enzyme 1, mitochondrial<br>FUM1_ARATH Fumarate hydratase 1, mitochondrial

GO:1901575	organic substance catabolic process	GO:0009056, GO:0071704	catabolic process, organic substance metabolic process	7.52	24	PLDA1_ARATH Phospholipase D alpha 1<br>ODO2A_ARATH Dihydrolipoyllysine-residue succinyltransferase component of 2-oxoglutarate dehydrogenase complex 1, mitochondrial<br>ODO2B_ARATH Dihydrolipoyllysine-residue succinyltransferase component of 2-oxoglutarate dehydrogenase complex 2, mitochondrial<br>GLO2C_ARATH Hydroxyacylglutathione hydrolase cytoplasmic<br>JAL28_ARATH Nitrile-specifier protein 1<br>XCP2_ARATH Cysteine protease XCP2<br>PRS6B_ARATH 26S proteasome regulatory subunit 6B homolog<br>PS12A_ARATH 26S proteasome non-ATPase regulatory subunit 12 homolog A<br>GDL18_ARATH GDSL esterase/lipase 22<br>BGL22_ARATH Beta-glucosidase 22<br>BGL21_ARATH Beta-glucosidase 21<br>GCSP2_ARATH Glycine dehydrogenase (decarboxylating) 2, mitochondrial<br>DCI1_ARATH Delta(3,5)-Delta(2,4)-dienoyl-CoA isomerase, peroxisomal<br>THIC2_ARATH Probable acetyl-CoA acetyltransferase, cytosolic 2<br>CINV2_ARATH Alkaline/neutral invertase CINV2<br>LGUC_ARATH Probable lactoylglutathione lyase, chloroplastic<br>GCSP1_ARATH Glycine dehydrogenase (decarboxylating) 1, mitochondrial

Table S4. List of proteins differentially abundant in roots of both *pldα1-1* and *pldα1-2* mutants (in comparison with control) containing mitochondrial targeting presequence as predicetd by MitoFates [73].

Sequence ID	Probability of presequence	Cleavage site (processing enzyme)	Net charge	Positions for TOM20 recognition motif (deliminated by comma)	Position of amphypathic alpha-helix	BHHPPP	BPHBHH	HBHHBb	HHBHHB	HHBPHH	HHBPHP	HHHBBH	HHHBPH	HHHHBB	HHPBHH	HPBHHP	PHHBPH
sp|Q9FLQ4|ODO2A_ARATH Dihydrolipoyllysine-residue succinyltransferase component of 2-oxoglutarate dehydrogenase complex 1	1	85(MPP), 86(Icp55*)	0.106	2-6,79-83	2-11-high	-	-	-	-	-	-	5.10	-	-	-	-	-
sp|Q8S528|AL2B7_ARATH Aldehyde dehydrogenase family 2 member B7	0.998	26(MPP)	0.192	93-97	4-14-high	-	-	-	-	-	-	-	-	-	-	-	-
sp|Q96251|ATPO_ARATH ATP synthase subunit O	0.996	35(MPP), 36(Icp55*)	0.143		1-10-low	-	-	-	-	11.16	-	-	-	-	-	-	10.15
sp|O04904|PYRC_ARATH Dihydroorotase	0.996	33(MPP)	0.182	52-56,64-68,86-90,90-94	1-10-high	-	-	-	-	1.6	-	-	-	-	-	-	-
sp|O04630|SYTM1_ARATH Threonine--tRNA ligase,	0.992	13(MPP)	0.308		2-13-high	-	-	-	-	-	-	-	-	-	-	-	-
sp|Q8H107|ODO2B_ARATH Dihydrolipoyllysine-residue succinyltransferase component of 2-oxoglutarate dehydrogenase complex 2	0.991	29(MPP)	0.172	2-6,7-11,78-82	2-11-high	-	-	-	-	-	20-25	5.10	19-24	-	-	-	-
sp|Q9SIU0|MAO1_ARATH NAD-dependent malic enzyme 1	0.989	22(MPP)	0.227	14-18,86-90	13-22-high	-	-	-	-	-	-	-	-	-	-	-	-
sp|P93033|FUM1_ARATH Fumarate hydratase 1	0.978	27(MPP), 28(Icp55*)	0.148	18-22,74-78,81-85	21-30-low	-	-	-	-	-	-	-	-	-	-	-	-
sp|P40941|ADT2_ARATH ADP,ATP carrier protein 2	0.977	29(MPP), 30(Icp55*)	0		10-19-high	-	-	-	-	-	-	-	-	-	-	-	-

sp|Q9LJL3|PREP1_ARATH Presequence protease 1	0.97	27(MPP)	0.185	17-21	19-28-high	-	-	-	17-22	-	1.6	-	-	-	-	-	-
sp|Q9FN42|CLPP2_ARATH ATP-dependent Clp protease proteolytic subunit 2	0.963	29(MPP), 30(Icp55*)	0.138	50-54	1-10-high	10.15	-	-	-	-	-	-	-	-	-	-	-
sp|Q42577|NDUS7_ARATH NADH dehydrogenase [ubiquinone] iron-sulfur protein 7	0.948	21(MPP)	0.143	61-65	4-13-low	-	-	-	-	-	-	-	-	-	-	-	-
sp|P93736|SYVM1_ARATH Valine--tRNA ligase	0.94	38(MPP)	0.132		4-13-high	-	-	-	-	-	-	4.9	-	3.8	-	-	-
sp|O49543|MNIF1_ARATH Cysteine desulfurase	0.923	26(MPP), 27(Icp55*)	0.231	2.6	6-16-high	-	-	-	-	-	-	-	-	-	1.6	2.7	-
sp|Q9LKA5|MORF8_ARATH Multiple organellar RNA editing factor 8	0.859	25(MPP), 26(Icp55*)	0.16		9-18-high	-	-	-	-	-	-	-	-	-	-	-	-
sp|F4JTE7|GPP1_ARATH (DL)-glycerol-3-phosphatase 1	0.726	46(MPP)	0.196	71-75	1-10-high	-	-	-	-	-	-	-	-	-	-	-	-
sp|Q9FHG2|RL322_ARATH 60S ribosomal protein L32-2	0.547	21(MPP)	0.333	72-76	11-20-high	-	14-19	16-21	-	-	-	-	-	-	-	-	-
sp|Q9LE82|RAGP1_ARATH RAN GTPase-activating protein 1	0.514	26(MPP)	0.154	84-88	1-12-high	-	-	-	-	-	-	-	-	-	-	-	-
sp|Q9XGM1|VATD_ARATH V-type proton ATPase subunit D	0.5	30(MPP)	0.133	49-53,81-85	15-24-low	-	-	-	-	-	-	-	-	-	-	-	-
sp|O80988|GCSP2_ARATH Glycine dehydrogenase (decarboxylating) 2	0.402	41(MPP)	0.171	4-8,13-17	9-18-high	-	-	-	-	-	-	-	-	-	-	-	-

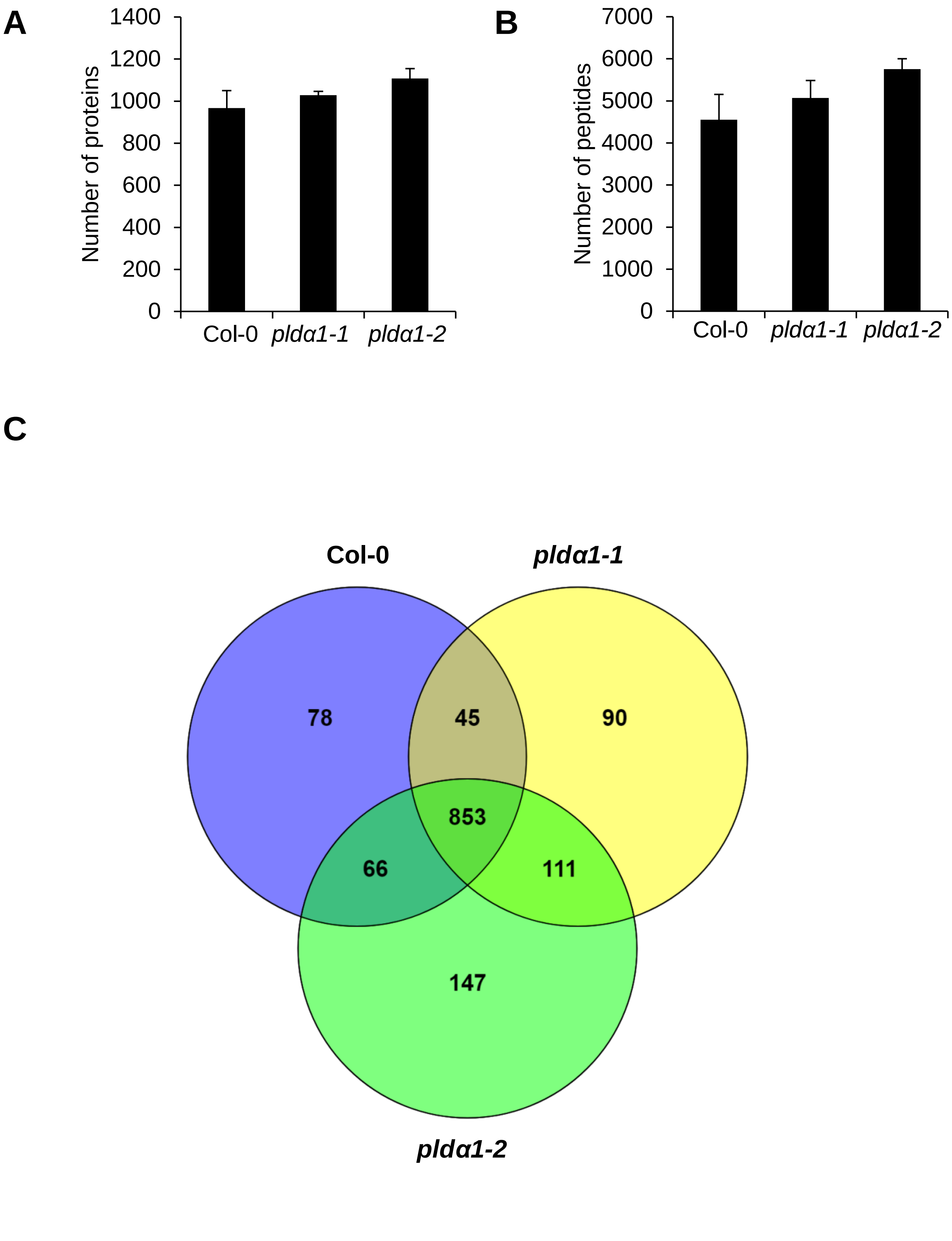

Figure S1. Evaluation of the total proteome of the studied Arabidopsis lines. (A) Graph showing the average number of identified proteins in the total proteomes of Col-0, *pldα1-1* and *pldα1-2* roots. (B) Graph showing the average number of identified peptides in Col-0, *pldα1-1* and *pldα1-2* roots. The errorr bars in (A) and (B) represent standard deviations across 4 biological replicates. (C) Venn diagram showing the overlapping and unique proteins identified in Col-0, *pldα1-1* and *pldα1-2* mutant roots. Proteins present at least two biological replicates were evaluated.

A

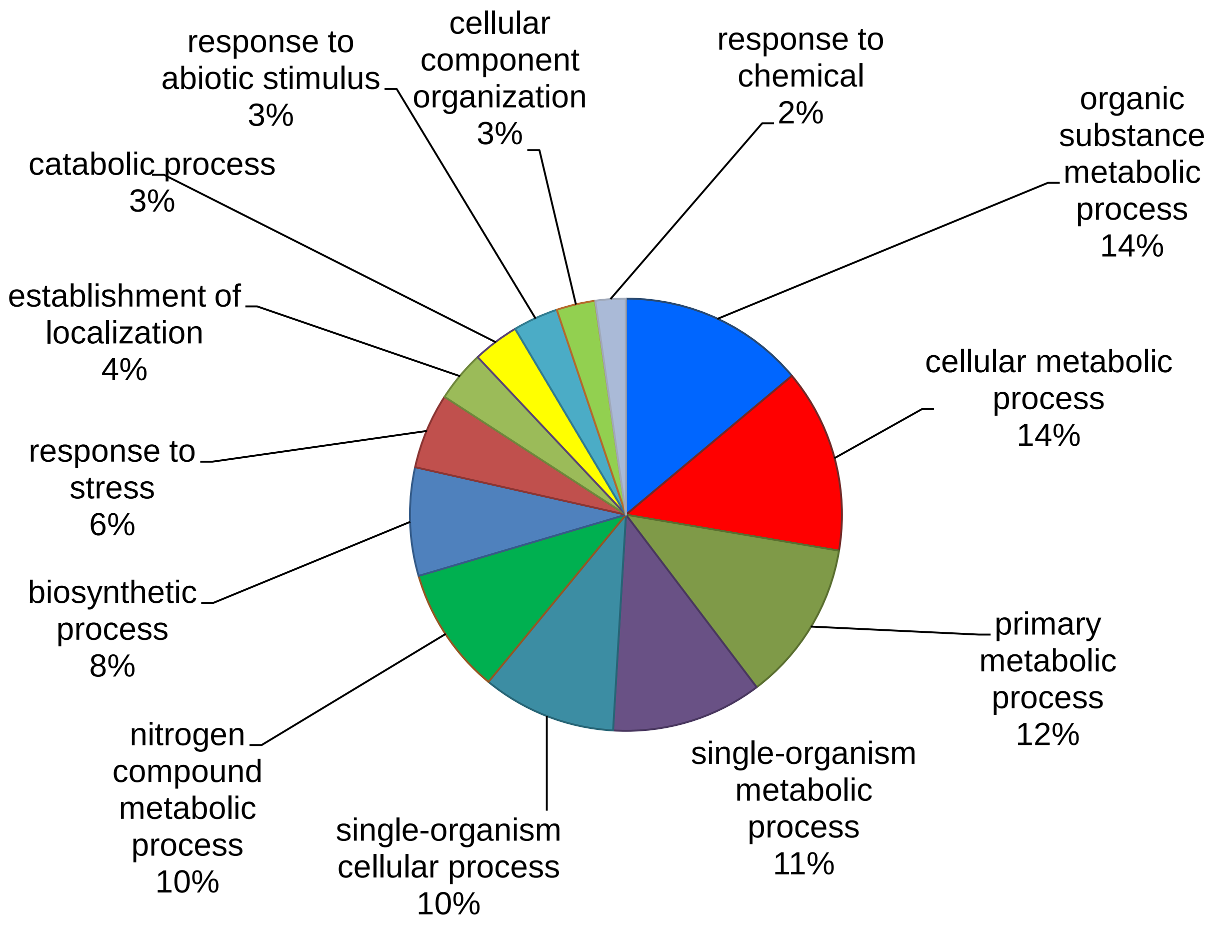

B

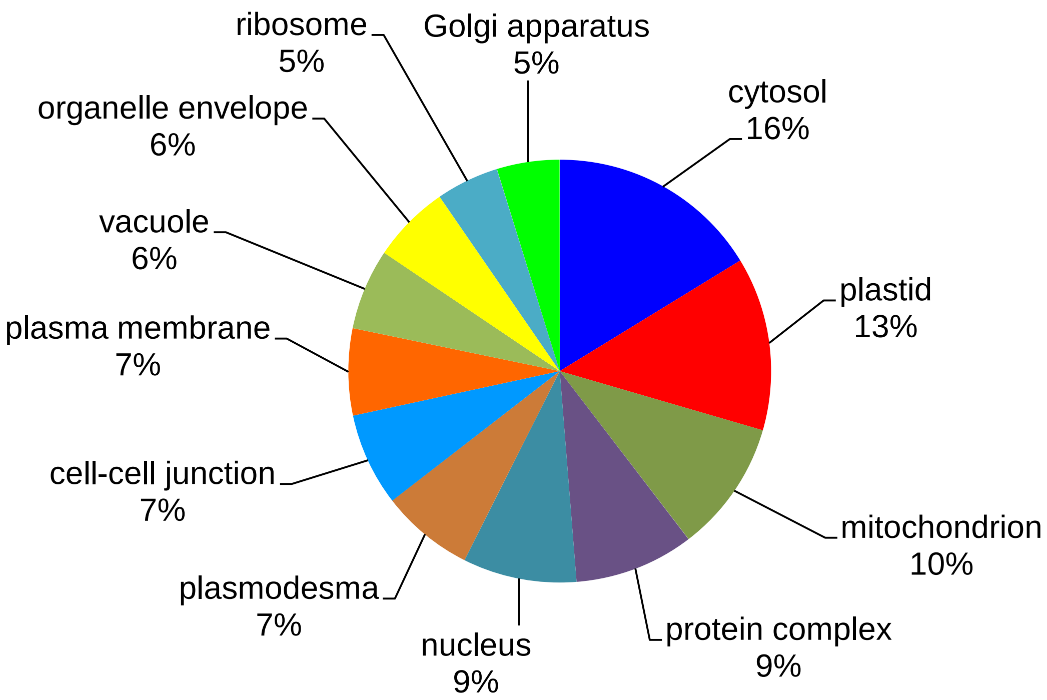

Figure S2 Functional classification of differentially abundant proteins found in roots of *pldα1-1* and *pldα1-2* mutants using Gene onthology annotation according to biological process (A; third level of onthology) and compartment (B).
